# Identification of a Potent Phosphoinositide 3‐Kinase Pan Inhibitor Displaying a Strategic Carboxylic Acid Group and Development of Its Prodrugs

**DOI:** 10.1002/cmdc.201700340

**Published:** 2017-08-31

**Authors:** Tracey Pirali, Elisa Ciraolo, Silvio Aprile, Alberto Massarotti, Alex Berndt, Alessia Griglio, Marta Serafini, Valentina Mercalli, Clarissa Landoni, Carlo Cosimo Campa, Jean Piero Margaria, Rangel L. Silva, Giorgio Grosa, Giovanni Sorba, Roger Williams, Emilio Hirsch, Gian Cesare Tron

**Affiliations:** ^1^ Dipartimento di Scienze del Farmaco Università degli Studi del Piemonte Orientale “A. Avogadro” Largo Donegani 2 28100 Novara Italy; ^2^ Department of Molecular Biotechnology and Health Sciences University of Torino Via Nizza 52 10126 Torino Italy; ^3^ MRC Laboratory of Molecular Biology Medical Research Council Cambridge CB2 0QH UK; ^4^ Department of Pharmacology, Ribeirão Preto Medical School University of São Paulo Avenida Bandeirantes 3900 14049-900 Ribeirão Preto Brazil; ^5^ Kither Biotech S.r.l. Molecular Biotechnology Center Via Nizza 52 10126 Torino Italy

**Keywords:** click chemistry, inflammation, nitrogen heterocycles, phosphoinositide 3-kinases, prodrugs

## Abstract

Activation of the phosphoinositide 3‐kinase (PI3K) pathway is a key signaling event in cancer, inflammation, and other proliferative diseases. PI3K inhibitors are already approved for some specific clinical indications, but their systemic on‐target toxicity limits their larger use. In particular, whereas toxicity is tolerable in acute treatment of life‐threatening diseases, this is less acceptable in chronic conditions. In the past, the strategy to overcome this drawback was to block selected isoforms mainly expressed in leukocytes, but redundancy within the PI3K family members challenges the effectiveness of this approach. On the other hand, decreasing exposure to selected target cells represents a so‐far unexplored alternative to circumvent systemic toxicity. In this manuscript, we describe the generation of a library of triazolylquinolones and the development of the first prodrug pan‐PI3K inhibitor.

## Introduction

**Figure 1:**
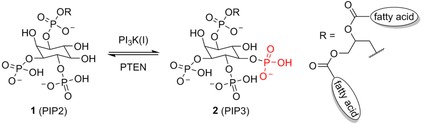
Class I PI3Ks phosphorylate the 3‐hydroxy group of phosphatidylinositol 4,5‐bisphosphates. PTEN=phosphatase and tensin homologue.

The discovery of phosphoinositide 3‐kinases (PI3Ks) dates back to 1985 when, with the seminal work of Cantley et al.,^1^ the specific lipid kinases able to phosphorylate the alcoholic group selectively at the 3‐position of phosphatidylinositols were reported. Three subfamilies of PI3Ks have been identified so far, referred to as classes I, II, and III. Class I is the most studied and comprises four isoforms, PI3Kα, PI3Kβ, PI3Kδ, and PI3Kγ, that share some structural features but that are distinct for protein domains, regulatory subunits, and activation mechanism.^2^ All class I PI3Ks can catalyze the in vivo conversion of phosphatidylinositol 4,5‐bisphosphate (**1**, PIP2) into phosphatidylinositol 3,4,5‐trisphosphate (**2**, PIP3) (Figure 1). PIP3 production induces the recruitment to the plasma membrane of 3‐phosphoinositide‐dependent protein kinase‐1 (PDK1), which in turn activates protein kinase B (Akt) through phosphorylation on Thr308.

**Figure 2:**
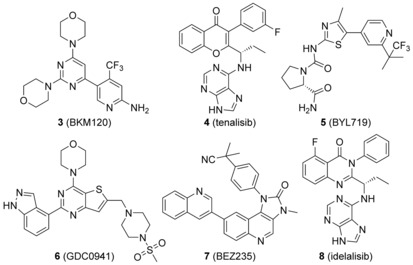
A selection of PI3K inhibitors in clinical trials, and the molecular structure of idelalisib.

Thirty years of basic research on PI3Ks have unequivocally demonstrated their involvement in a plethora of biological processes such as cell growth, proliferation, differentiation, and motility.^3^ Furthermore, hyperactivation of the PI3K/Akt signaling pathway has been linked to different pathologies, such as cancer and autoimmune diseases.^4^, ^5^ Studies performed with the use of animal models and PI3K inhibitors have established PI3Kα and PI3Kβ as promising targets for the treatment of human cancer.^6^ Concurrently, the central role of PI3Kγ and PI3Kδ in leukocyte biology sustains their inhibition as a promising therapeutic approach in a variety of inflammatory diseases.^5^ These findings have prompted both academia and industry research to develop inhibitors targeting all PI3K isoforms. Most of these molecules act by antagonizing the binding of ATP to the PI3K catalytic pocket. To date, hundreds of pan‐ and tens of isoform‐selective PI3K inhibitors have been discovered, and some of them are in clinical evaluation for tumor treatment (Figure 2).^6^, ^7^, ^8^ These efforts culminated in 2014 with the FDA approval of idelalisib (**8**), a PI3Kδ‐selective inhibitor for the treatment of patients with relapsed follicular B‐cell non‐Hodgkin lymphoma or relapsed small lymphocytic lymphoma (SLL).^9^

A more accurate analysis of the structure of already‐reported PI3K inhibitors^10^ highlighted the lack of molecules displaying a carboxylic acid group that we here report to be fundamental for binding to the PI3 kinase structural motif. Furthermore, this functional group can undergo chemical modifications with the intent to develop useful prodrugs. Indeed, this approach might circumvent nonspecific targeting and avoid systemic side effects associated with the use of a pan‐PI3K inhibitor, especially if proposed for topical use. Therefore, in the present manuscript we report the discovery of **37**, a potent pan‐PI3K inhibitor displaying a strategic and pivotal carboxylic acid group, which can be esterified to provide prodrugs. The synthesis of **37** and the metabolic stability of its prodrugs, along with their full biological characterization, are discussed herein.

## Results and Discussion

### Chemistry

#### Design, synthesis, and enzyme inhibition

**Figure 3:**
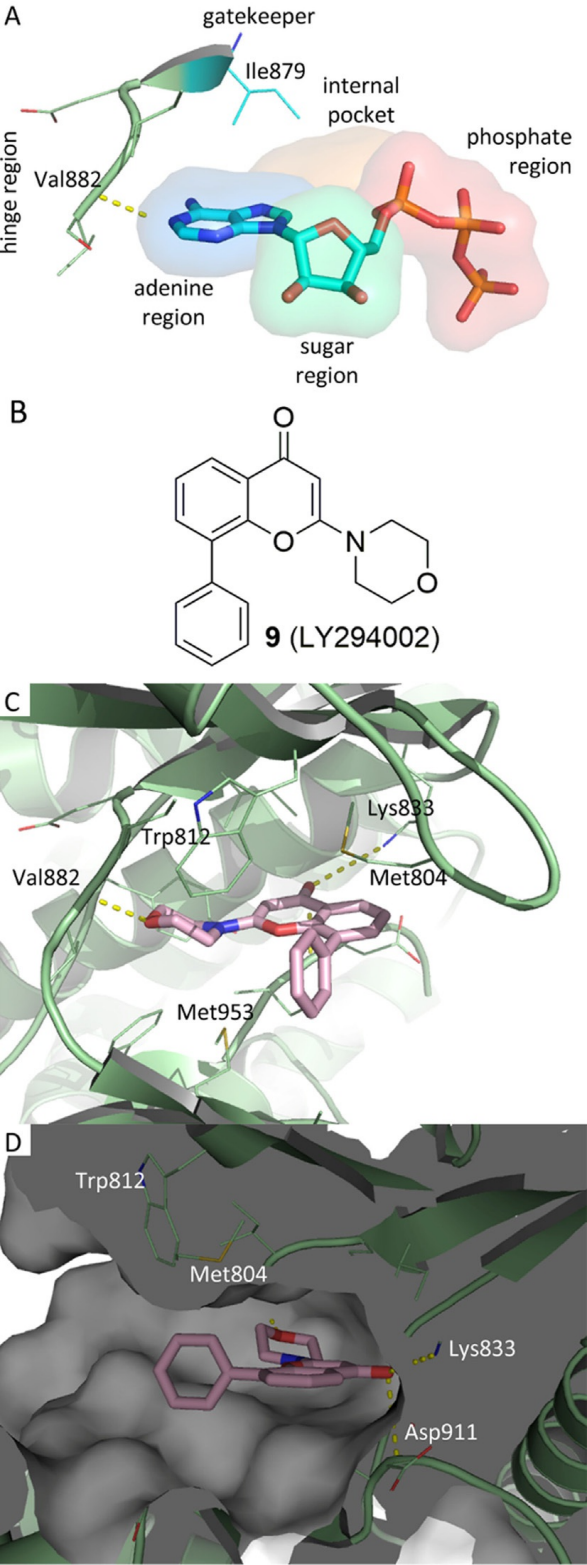
X‐ray structures of pig PI3Kγ in complex with a) ATP (PDB ID: 1E8X) and c, d) **9** (PDB ID: 1E7V). b) 2 D structure of **9**. Protein is shown in pale‐green cartoon; ligands atoms are shown as sticks. Hydrogen‐bonding interactions are plotted as yellow dotted lines. The **9**–protein complex is shown from two different points of view: b) front and c) side.

On the basis of the considerations discussed above, we decided to start our project by modifying compound LY294002 (**9**, Figure 3), one of the first pan‐PI3K inhibitors discovered.^11^ This quercetin analogue competes with ATP in the ATP‐binding site of PI3Ks, with an IC_50_ value of ∼0.5 μm against all of the isoforms. The X‐ray structure of **9** bound to PI3Kγ is also available,^12^ and it shows that the morpholino ring partially overlaps the volume occupied by the adenine in the ATP–enzyme complex (Figure 3).

A hydrogen bond is formed between the morpholino oxygen atom and the backbone amide group of residue Val882 of the hinge region, and this bond mimics the interaction that ATP establishes with the enzyme. There is also a putative hydrogen bond between Lys833 and the carbonyl group. The phenyl ring is located in a pocket, usually occupied by the ribose of ATP, formed by Met804 and Trp812 on one side and Met953 on the other, and it protrudes toward the solvent‐accessible part of the binding pocket. Interestingly, **9** does not extend into the phosphate‐binding region.

**Figure 4:**
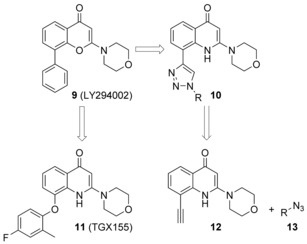
LY294002 (**9**), TGX155 (**11**), and design of new analogues by a click‐chemistry approach.

We therefore reasoned that the phenyl ring could be replaced with a 1,4‐disubstituted 1,2,3‐triazole by a click‐chemistry approach.^13^ In this case, the heterocycle ring would behave as a mere linker, delivering an extra site R (Figure 4), that might establish additional interactions with the close amino‐acid residues, either enhancing the potency of **9** or imparting selectivity. Indeed, it is important to highlight that the rim of the binding site of class I PI3Ks is the region of the enzyme for which there is major variability in the amino‐acid composition among all the four isoforms,^14^ and therefore, it is more amenable to be targeted in the search for selectivity. Owing to the versatility and functional‐group tolerance of the Fokin–Sharpless reaction,^15^ a combinatorial approach was exploited that would allow exploration of a vast array of different functional groups in the R portion (Figure 4). It is important to highlight that modification of the phenyl ring of **9** was already attempted and described. For example, TGX155 (**11**), in which the phenyl ring is replaced with a 4‐fluoro‐2‐methylphenoxy moiety, was shown to be a selective, potent inhibitor of the β isoform of PI3K and a potential therapeutic agent for cardiovascular diseases.^16^ This example corroborates our hypothesis to replace the phenyl ring of **9**. In our specific case, we opted to diversify the chemical nature of the R group by exploiting different azides **13**. The azides were preferred as decorating groups rather than alkynes because their chemical preparation, starting from commercially available amines, is easier than the more challenging synthesis of alkynes.

**Scheme 1:**
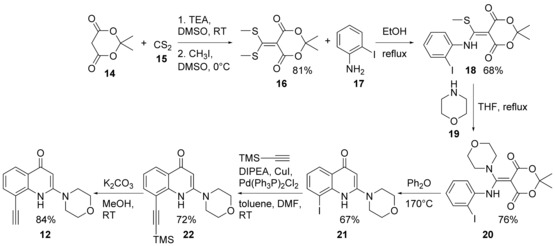
Synthesis of alkyne **12**. TEA=triethylamine, TMS=trimethylsilyl, DIPEA=*N*,*N*‐diisopropylethylamine.

Once the goal was fixed, we proceeded with the synthesis of the pivotal alkyne. The preparation of **12** started with the reaction between Meldrum's acid (**14**) and CS_2_ (**15**). The intermediate was then methylated in situ to give **16**.^17^ Subsequently, this compound underwent a first nucleophilic displacement with 2‐iodoaniline (**17**) at reflux to give **18**, which was then treated with morpholine (**19**) in THF at reflux to give the second nucleophilic substitution to lead to compound **20**. Heating compound **20** in diphenyl ether at 170 °C triggered intramolecular aromatic nucleophilic substitution followed by a decarboxylation reaction to afford iodo intermediate **21**. Sonogashira coupling with trimethylsilyl acetylene gave **22** and subsequent deprotection under basic conditions afforded desired alkyne **12** (Scheme 1).

**Scheme 2:**
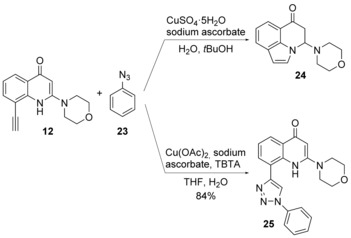
Optimization of the click reaction.

Once alkyne **12** was obtained, a combinatorial parallel synthesis with 30 azides was performed. To this point, before starting the construction of the library, a preliminary model reaction with phenyl azide (**23**) was attempted, which revealed that under the standard conditions of the Fokin–Sharpless click reaction (CuSO_4_ **⋅**5 H_2_O, sodium ascorbate, *t*BuOH/H_2_O) unexpected compound **24** was obtained instead of desired triazole **25**. It was reasonable to suppose that copper triggered intramolecular cyclization between the N1 atom and the triple bond, with the formation of a 6‐oxopyrrolquinolinic ring (Scheme 2).^18^ In light of this event, we reasoned that in the presence of an already‐chelated copper ion, this transformation might be suppressed. The ligand tris[(1‐benzyl‐1*H*‐1,2,3‐triazol‐4‐yl)methyl]amine (TBTA, also known as the Chan ligand) was our first choice. It was previously shown that TBTA was indeed able to stabilize the oxidation state of the copper(I) ion by encapsulating it in its structure and preventing other interactions.^19^ The use of the Chan ligand was successful, and under optimized reaction conditions (THF/H_2_O, Cu(OAc)_2_, sodium ascorbate, TBTA), desired 1,2,3‐triazole **25** was obtained in excellent yield (Scheme 2).

With this procedure in hand, we proceeded with our combinatorial strategy. The azides were selected to introduce both electron‐donating and electron‐withdrawing groups, hydrogen‐bond donors and acceptors, and ionizable functions. They were already present in our database or prepared according to standard procedures. The feasibility of this approach was demonstrated by precipitation of the resultant products, which were purified by filtration and subsequent water/diethyl ether washes. This procedure resulted in the formation of 30 compounds, which were submitted to MS and ^1^H NMR spectroscopy analysis to verify their purity (>95 %) and structural identity. Then, the 1,2,3‐triazoles were evaluated for their ability to inhibit PI3K isoforms of class I at a fixed concentration of 1 μm, as shown in Table 1. The synthesized 1,2,3‐triazoles along with their ability to inhibit PI3K isoforms at 1 μm concentration are reported. Possibly, a minute concentration of copper salts might have precipitated with the products and might have been retained in the purified compounds, and so, we also evaluated the PI3K inhibitory activity of these salts. Neither copper(II) acetate nor copper(I) iodide displayed significant inhibition of PI3Ks up to 10 μm.

**Table 1:** Inhibitory activity against different PI3K isoforms.^[a]^

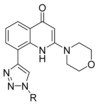					
Compd	R	PI3K isoform			
		α	β	γ	δ
**25**	phenyl	+	−	−	+
**26**	3‐hydroxyphenyl	−	−	−	−
**27**	3‐hydroxy‐4‐methoxyphenyl	−	−	−	−
**28**	4‐chlorophenyl	−	−	−	−
**29**	4‐(trifluoromethoxy)phenyl	−	−	−	−
**30**	4‐methoxy‐3‐nitrophenyl	−	−	−	−
**31**	2‐(4‐(methoxycarbonyl)benzyl	−	−	−	−
**32**	4‐aminophenyl	−	−	−	−
**33**	4‐(cyanomethyl)phenyl	−	−	−	−
**34**	naphthalen‐2‐yl	−	−	−	−
**35**	3‐methoxyphenyl	−	−	−	+
**36**	quinolin‐3‐yl	−	−	−	−
**37**	3‐carboxybenzyl	+	+	+	+
**38**	5‐acetamido‐2‐methoxyphenyl	−	−	−	−
**39**	5‐methoxy‐2‐(trifluoromethyl)phenyl	−	−	−	−
**40**	4‐hydroxy‐2‐methylphenyl	−	−	−	−
**41**	2‐(hydroxymethyl)phenyl	−	−	−	−
**42**	4‐(methoxycarbonyl)phenyl	−	−	−	−
**43**	3,4‐dimethoxyphenyl	−	−	−	−
**44**	4‐methoxyphenyl	−	−	−	−
**45**	4‐carboxyphenyl	+	+	+	+
**46**	benzo[*d*][1,3]dioxol‐5‐yl	−	−	−	−
**47**	3,5‐dimethoxyphenyl	−	−	−	−
**48**	benzyl	−	+	+	+
**49**	3‐(methoxycarbonyl)phenyl	−	−	−	−
**50**	4‐sulfamoylphenyl	−	−	−	−
**51**	2‐methoxybenzyl	−	−	−	−
**52**	3‐carboxyphenyl	−	−	−	−
**53**	naphthalen‐1‐yl	−	−	−	−
**54**	2‐hydroxyphenyl	+	−	−	+

[a] PI3K inhibition determined at 1 μm with at least triplicate determinations in multiple experiments; ′+′ refers to those compounds able to inhibit PI3K isoform activity by more than 50 %.

Only those compounds able to suppress the kinase activity by 50 % of at least one isoform (i.e., **25**, **35**, **37**, **45**, **48**, and **54**) at 1 μm were resynthesized, purified by column chromatography, and re‐evaluated to obtain a precise IC_50_ value.

**Figure 5:**
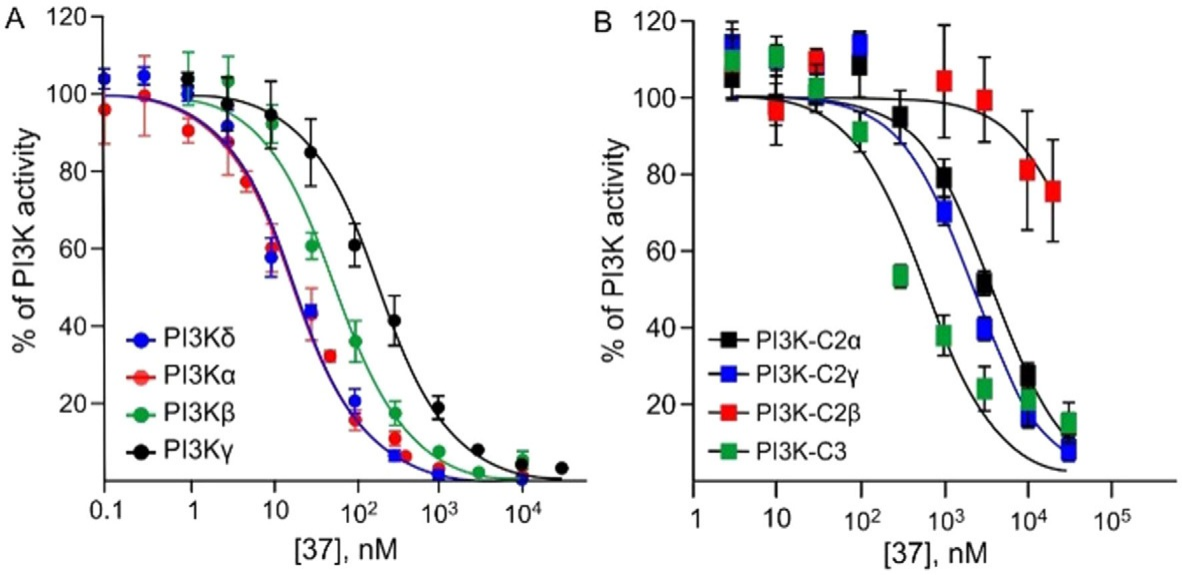
Inhibition profile of **37** against a) class I and b) classes II and III PI3Ks. Each PI3K isoform (30 ng) was incubated with various concentrations of **37** in the presence of ATP (10 μm for PI3Kα, PI3Kβ, PI3Kγ, PI3Kδ, PI3K‐C2α, and PI3K‐C3; 100 μm for PI3K‐C2γ and PI3K‐C2β).^20^ ATP loss and consequent ADP production were measured though a luminometric assay. The residual PI3K activity after treatment with different concentrations of **37** was calculated as a percentage of the untreated control (recombinant protein+DMSO). The dose–response curve was obtained by plotting the PI3K activity [%] versus the logarithms of the inhibitor concentrations. Data are the mean±SD of at least two independent experiments each performed in triplicate.

Compound **37** emerged from the screening as the most‐potent inhibitor with higher selectivity against PI3Kα (IC_50_=18 nm) and PI3Kδ (IC_50_=19 nm) than against PI3Kβ (IC_50_=59 nm) and PI3Kγ (IC_50_=186 nm). Its inhibitory nature was tested on other PI3Ks of classes II and III (Table 2 and Figure 5), demonstrating its selectivity over class I isoforms.

**Table 2:** IC_50_ values of **25**, **35**, **37**, **45**, **48** and **54** on different PI3K isoforms.^[a]^

	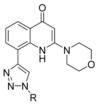						
	IC_50_ [μm]						
	TGX155	**25**	**35**	**37**	**45**	**48**	**54**
PI3Kα	>10	0.74±0.31	1.3±0.7	0.018±0.004	0.32±0.2	2.65±0.8	0.26±0.12
PI3Kβ	0.096±0.007	5.5±2.5	9.3±5.8	0.059±0.015	0.30±0.2	0.10±0.04	1.1±0.5
PI3Kγ	9.40±1.2	3.0±1.0	4.2±2.1	0.186±0.043	0.30±0.2	1.0±0.5	1.4±0.44
PI3Kδ	0.41±0.13	0.61±0.27	0.81±0.36	0.019±0.004	0.17±0.1	0.47±0.26	0.35±0.17
PI3Kc2α	n.d.	n.d.	n.d.	3.7±1.1	n.d.	n.d.	n.d.
PI3Kc2β	n.d.	n.d.	n.d.	>20	n.d.	n.d.	n.d.
PI3Kc2γ	n.d.	n.d.	n.d.	2.8±0.7	n.d.	n.d.	n.d.
PI3Kc3	n.d.	n.d.	n.d.	0.59±0.2	n.d.	n.d.	n.d.

[a] Values are the means of at least three determinations in multiple experiments, with 95 % confidence intervals; n.d.: not determined.

These multiple experiments highlight the pivotal role played by the carboxylic acid if positioned in the *meta* position on the benzyl ring in increasing the potency of this class of compounds. Therefore, starting from **37**, we decided to replace the 3‐methylbenzoic acid group with three linear aliphatic carboxylic acids, namely, propionic acid, butyric acid, and pentanoic acid (i.e., to give compounds **55**, **56**, and **57**, respectively), and the carboxyl moiety with its canonical bioisosteres such as sulfonamide (i.e., compound **58**); primary (i.e., compound **59**), secondary (i.e., compounds **60** and **61**), and tertiary (i.e., compound **62**) amides; and finally tetrazole (i.e., compound **63**). All the attempted substitutions were detrimental to inhibition activity (Table 3).

**Table 3:** IC_50_ values of analogues of **37** against PI3Kα.^[a]^

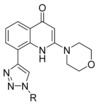		
Compd	R	IC_50_ [μm]
**55**	3‐carboxypropyl	>1
**56**	4‐carboxybutyl	>1
**57**	5‐carboxypentyl	>1
**58**	3‐[(methylsulfonyl)carbamoyl]benzyl	>1
**59**	3‐carbamoylbenzyl	0.800±0.74
**60**	3‐(benzylcarbamoyl)benzyl	0.140±0.09
**61**	3‐(cyclopropylcarbamoyl)benzyl	0.250±0.08
**62**	3‐(diethylcarbamoyl)benzyl	0.290±0.08
**63**	3‐(1*H*‐tetrazol‐5‐yl)benzyl	>1

[a] Values are the means of at least three determinations in multiple experiments, with 95 % confidence intervals; n.d.: not determined. See the Experimental Section for the synthesis of compound **63**.

**Figure 6:**
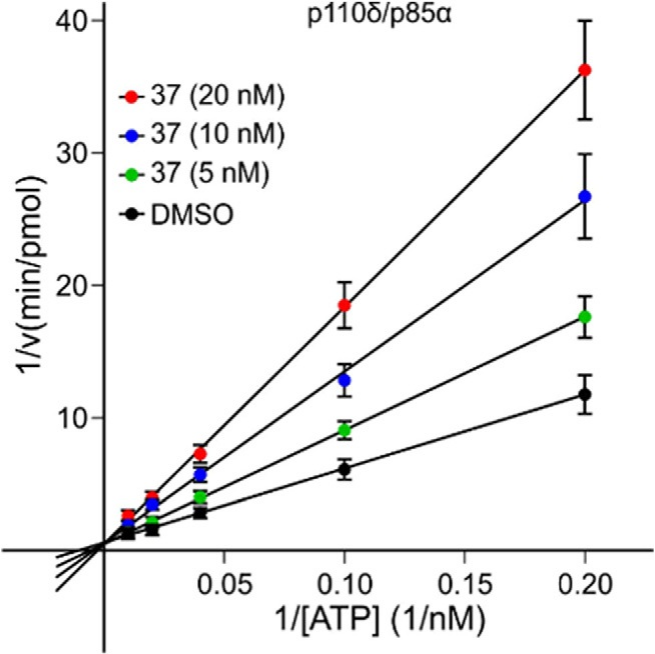
Inhibition mode of **37** against PI3Kδ isoform. Lineweaver–Burk plot, 1/*v* versus 1/[ATP]. The inhibition assay was performed by using varying concentrations of ATP (5, 10, 25, 50, and 100 μm) and fixed concentrations of PI3Kδ recombinant protein (2.4 μg mL^−1^) and lipids (1 mg mL^−1^ PI/PS lipid micelles mixture) in the presence or absence of different concentrations of **37** (5, 10, and 20 nm).

We then proceeded to evaluate the ATP competitive inhibitory nature of **37** through an enzymatic kinetic study by using an in vitro assay to measure the PI3Kδ activity at various concentrations of ATP and **37**. As shown in Figure 6, Lineweaver–Burk plot analysis revealed that **37** acts as an ATP‐competitive inhibitor for PI3Kδ, as the plots (straight lines) intersect on the 1/*v* axis (*v*=rate of ADP production).

**Figure 7:**
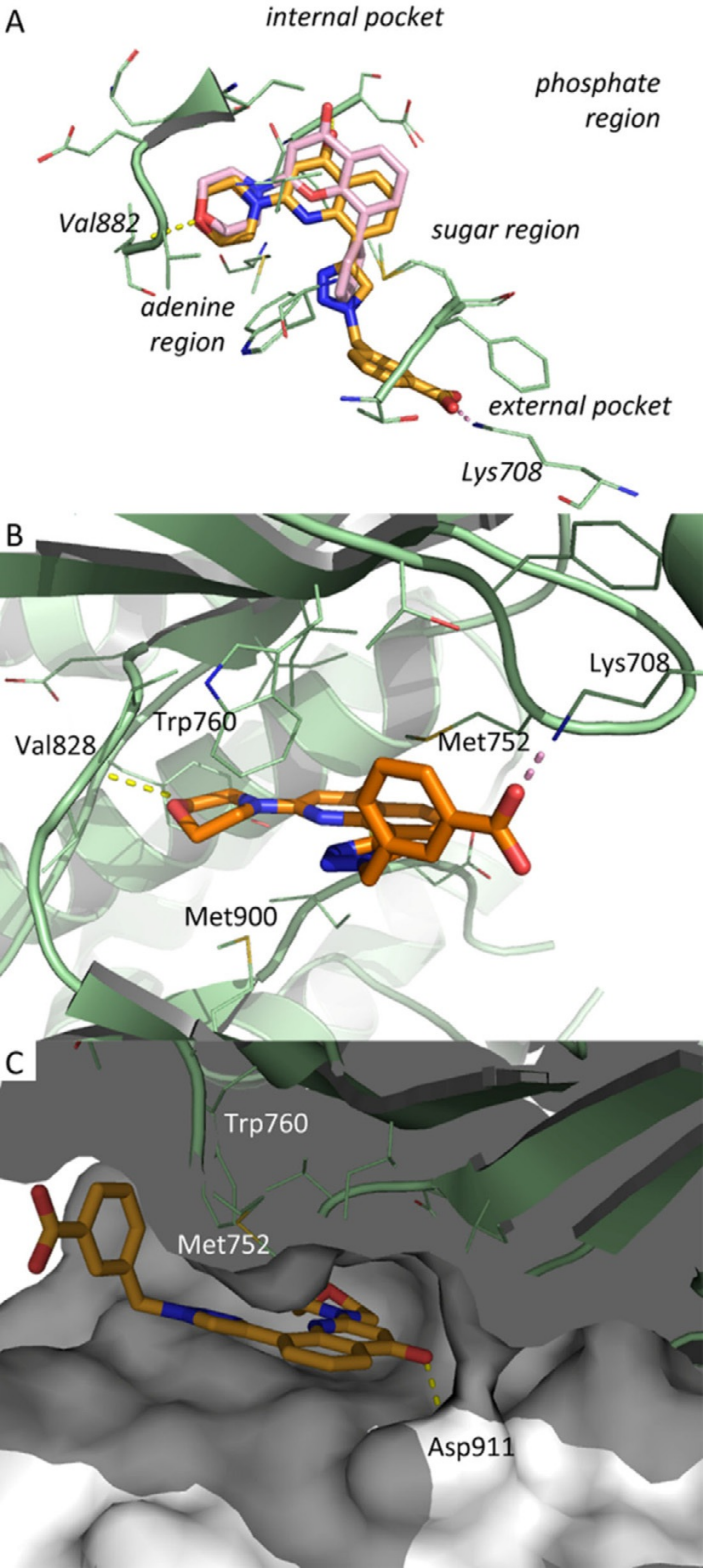
X‐ray structures of murine PI3Kδ in complex with **37** (PDB ID: 5NGB). Protein is shown in pale‐green cartoon; ligand is shown as sticks with carbon atoms depicted in orange. Hydrogen‐bonding and ionic interactions are plotted as yellow and pink dotted lines, respectively. The ligand–protein complex is shown from different points of view: a) top with the crystal structure of **9** (PDB ID: 1E7V) superposed as pink sticks, b) front, and c) side.

To investigate the binding of **37** in the enzyme active site, the crystal structure of **37** bound to the murine δ isoform of PI3K was resolved (see the Supporting Information for technical details). Compound **37** binds in the ATP binding site in a canonical mode (Figure 7), as described for other type I kinase inhibitors. In detail, the morpholine ring establishes a typical hydrogen bond with Val882 in the hinge region, analogously to **9**; the quinolone ring is in a central pocket with an orientation very similar to that of **9** (Figure 7 a), and the carbonyl group is involved in a putative hydrogen‐bonding interaction with Asp911. The 1,2,3‐triazole appears as fundamental to orientate the carboxylic acid group properly. Indeed, the X‐ray structure of **9** shows a perpendicular orientation of the phenyl ring to the central core, whereas **37** is characterized by coplanar orientation of the 1,2,3‐triazole to the quinolinone core. This different spatial disposition allows for a pivotal ionic salt‐bridge interaction between the carboxyl moiety and Lys708, which is consistent with the observed potent inhibitory activity.

### Biological assays

#### Cellular inhibitory activities

**Figure 8:**
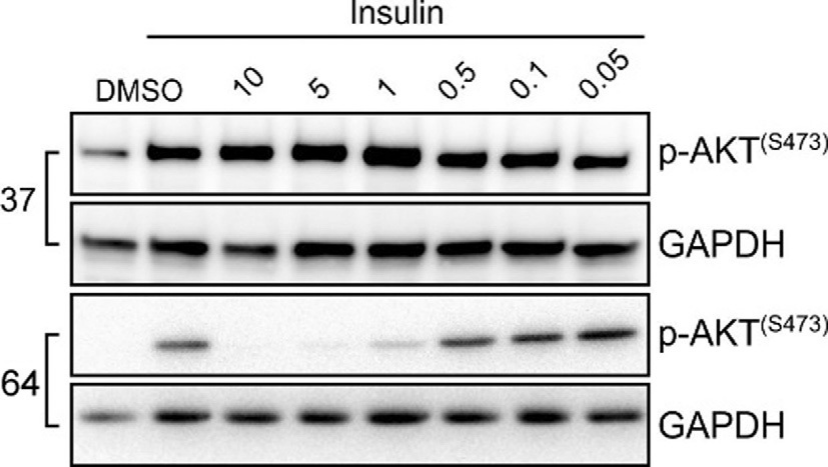
Treatment with **64** prodrug inhibited PI3K/Akt signaling. NIH3T3 cells were harvested for 12 h and were treated with the indicated concentrations of **37** and **64**. Cells were next stimulated with 1 μm insulin for 5 min, and total cell lysates were prepared. Immunoblot analysis was conducted for the expression levels of p‐AKT and GAPDH. The immunoblot is representative of three independent experiments.

After identifying **37** as a promising candidate, we started to evaluate it in cell‐based assays to define its inhibitory activity on the PI3K signaling pathway. Therefore, we selected an in vitro insulin model to assess the inhibitory effect of **37** in PI3K signaling. NIH3T3 cells were treated with different concentrations of **37**, stimulated with insulin, and the amount of phosphorylated Akt was detected. Nonetheless, as shown in Figure 8, **37** did not affect the PI3K signaling pathway, as it did not decrease Akt phosphorylation.

We therefore reasoned that the lack of activity of **37** in cell‐based experiments could be ascribed to its inability to cross cell membranes owing to the increased polarity imparted by the ionized carboxylic acid. For this reason, we prepared corresponding methyl ester **64**. A lipid kinase assay confirmed that **64** was not able to inhibit PI3Kα activity at 100 nm (see the Supporting Information), which confirmed the crucial role played by the carboxyl group in the interaction with PI3Ks. Nevertheless, given its hydrophobic nature, it was conceivable that the methyl ester could cross the membrane and, after intracellular enzymatic hydrolysis, explicate its inhibitory activity. To demonstrate that **64** could act as a prodrug, we treated NIH3T3 cells with different concentration of **64**, and we then analyzed Akt phosphorylation after insulin stimulation.

As shown in Figure 8, methyl ester **64** was able to inhibit the PI3K‐signaling pathway in a dose‐dependent manner.

**Figure 9:**
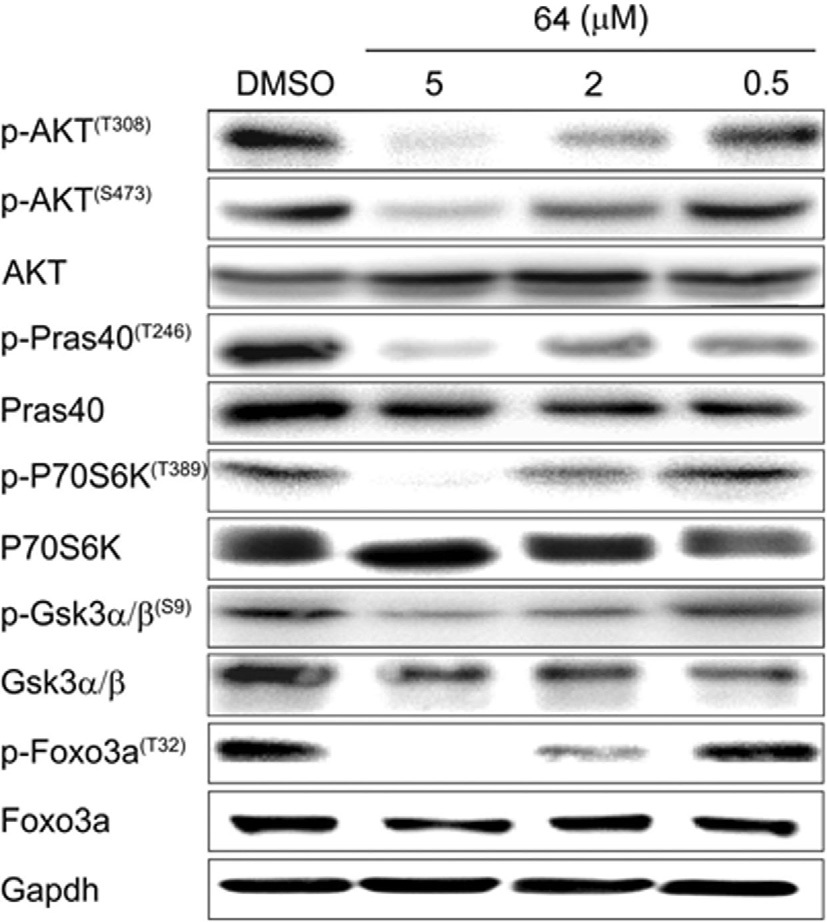
Treatment with **64** dose dependently inhibited PI3K/Akt signaling in PC3 cells. PC3 cells were treated with the indicated concentrations of **64** for 12 h. At the end of treatment, cells were harvested and total cell lysates were prepared. Immunoblot analysis was performed to analyze the expression levels of p‐Akt (T308 and S473), p‐PRAS40 (T246; a target of Akt), as well as the levels of p‐p70S6K (T389), p‐GSK3α/β (S9), and p‐FOXO3A (T32). The figure is representative of at least three independent experiments.

To determine whether **64** could inhibit PI3K/Akt in tumor cells, in which the PI3K signaling is hyperactive, PC3 prostatic tumor cells were incubated with different concentrations of **64** (Figure 9). Treatment with **64** attenuated, in a dose‐dependent manner, the levels of p‐Akt, as well as the levels of some Akt effectors such us p‐PRAS40, p‐p70S6K, p‐GSK3α/β, and p‐FOXO3A.

**Figure 10:**
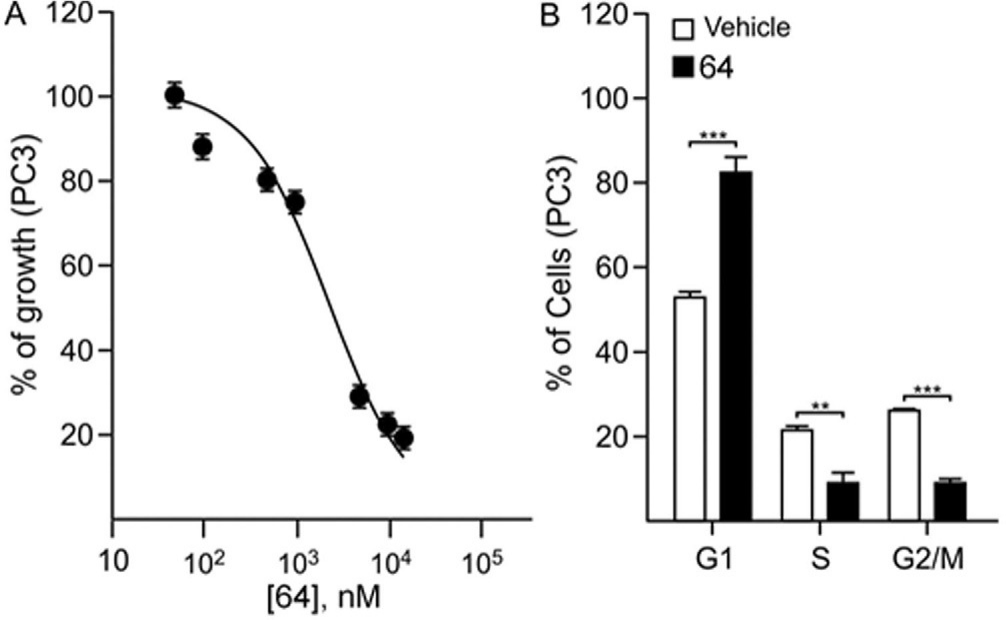
Compound **64** decreased proliferation and induced cell‐cycle arrest. a) Proliferation curve of PC3 cells. Cell proliferation was analyzed by using MTT cell‐proliferation assay kits. The growth was calculated as the percentage of the untreated control and a dose–response curve was generated by plotting the percentage of growing cells versus the logarithm of inhibitor concentration. The curve represents the average of three independent experiments. b) PC3 were treated with 10 μm **64** for 48 h. At the end of treatment, cells were washed with 1× phosphate‐buffered saline and were stained with propidium iodide. The percentage of cells in the G1/G0, S, and G2/M phases was determined by flow cytometry. Columns represent the mean of three independent experiments; bars represent the SEM; **p<0.01, ***p<0.001.

We next determined the effect of **64** on cell proliferation, cell cycle, and apoptosis. As shown in Figure 10 a, 72 h of treatment with **64** dose‐dependently decreased proliferation of PC3 cells. In addition, after 48 h of treatment, **64** was able to induce cell‐cycle arrest by significantly increasing the percentage of cells in the G0–G1 phase with concomitant reduction in the percentage in the S and G2/M phases (Figure 10 b). On the other hand, **64** did not show any effect in inducing cell apoptosis (see the Supporting Information).

#### Prodrug selection and metabolic stability

The opportunity to generate different prodrugs by acting on the carboxylic acid group stimulated the design and synthesis of a panel of different esterified products. It is important to highlight that by browsing the literature data on PI3K inhibitors it emerged that only one other reported PI3Kα inhibitor displayed a carboxylic acid, but in this case, the corresponding methyl ester was still able to inhibit the PI3 kinases and was only four times less active than the corresponding carboxylic acid analogue (IC_50_ on PI3Kα: 46.0 versus 10 nm).^21^

Twelve esters were prepared (see the Experimental Section for their synthesis and the Supporting Information for chromatographic methods) and evaluated in terms of metabolic stability. For this task, a combined approach based on microsomal and plasmatic stability assays was exploited considering the relevance of the plasmatic and hepatic tissues in esterase activity. Microsomal stability of the selected esters was evaluated by monitoring the disappearance of the substrate incubated in rat liver microsomes (RLM) at 50 μm concentration, in the presence of NADPH. In Table 4, the percentage of the remaining substrate, after incubation (*t*=60 min), with respect to the initial amount is reported. Overall, the compounds underwent a relevant metabolic transformation, and the substrate depletion ranged from 0 (for **76**) to 100 % (for **70**). This is mainly due to hydrolysis of the ester function, whereas the metabolites arising from the oxidation catalyzed by the NADPH‐dependent monooxygenase system were negligible from a quantitative point of view. Indeed, the main metabolite formed during the incubation of the esters was corresponding carboxyl derivative **37**. Furthermore, the addition of NADPH did not significantly increase the oxidative metabolic transformation (see Figure S3 in Supporting Information). As expected, the aliphatic esters were found to be hydrolytically labile, and ethyl ester **67** was less stable than methyl ester **64**,^22^ whereas long, linear alkanoates **75** and **76** and branched esters **69** and **74** showed greater stability. Moreover, whereas CH_2_‐pyridin‐4‐yl ester **70** was completely metabolized, corresponding benzyl derivative **65** and piperonyl derivative **72** were more hydrolytically stable, and their residual substrates were 53 and 83 %, respectively. The hydrolytic stability of the selected esters was also evaluated by monitoring the depletion of the substrate incubated at a 50 μm concentration in mouse plasma over a period of 30 min. As expected, the plasmatic metabolic fate of the esters was characterized by the formation of **37** as the only metabolite (see Figure S4). The rate constant *k* [min^−1^], derived for the substrate peak area versus time curve (single‐exponential decay equation—GraphPad Prism software, Inc., San Diego, CA (USA); see Figure S5) was used to calculate the in vitro half‐life *t*_1/2_ [min], and the values are reported in Table 4. Except for branched ester **69** and long, linear ester **76**, which were inert to the mouse plasma hydrolases, the other compounds underwent relevant hydrolytic cleavage with *t*_1/2_ values ranging from <1 to 15 min. In detail, short‐linear esters **67** and **73** in addition to benzyl derivative **65**, CH_2_‐pyridin‐4‐yl derivative **70**, and piperonyl derivative **72** were rapidly hydrolyzed, and their *t*_1/2_ values were less than 1 min (see the Supporting Information).

**Table 4:** Synthesized prodrugs, metabolic stability (based on LC–UV analysis), and effect on PC3 proliferation.^[a]^

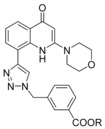				
Compd	R	Residual microsomal substrate [%] (*t*=60 min)	Mouse plasma *t*_1/2_ [min]	Cell proliferation
**64**	Me	20	13.3	active
**65**	Bn	53	<1	active
**66**	*i*Pr	16	15.1	active
**67**	Et	24	4.5	active
**68**	Bu	53	<1	active
**69**	*t*Bu	79	N.C.	inactive
**70**	CH_2_‐pyridin‐4‐yl	0	<1	active
**71**	(CH_2_)_2_‐morpholinyl	18	2.5	active
**72**	piperonyl	83	<1	active
**73**	isopentyl	39	<1	active
**74**	1‐methylbutanyl	60	4.2	inactive
**75**	(CH_2_)_10_Me	80	4.4	inactive
**76**	(CH_2_)_17_Me	100	N.C.	inactive

[a] N.C.: not calculable as hydrolytically stable. Active: able to reduce proliferation to an extent greater than 50 %; inactive: able to reduce proliferation to an extent less than 50 %.

The biological activity of each prodrug was tested by analyzing their ability to decrease PC3 proliferation as a rapid test to evaluate the PI3Ks inhibitory activity after hydrolysis in a cell‐based assay. PC3 cells were incubated with 10 μm of each prodrug: compounds able to decrease proliferation to an extent more than 50 % were defined as active, whereas those reducing to an extent less than 50 % were considered inactive. The choice of using such a cut‐off was due to the intent to identify rapidly which prodrug could be hydrolyzed in the assay to release the active compound. As expected, a good correlation between rate of hydrolysis and cellular activity was observed, and esters **64**–**68** and **70**–**73** were determined to be good candidates for topical use. We decided to focus our attention on ester **64**, as its half‐life was a good compromise for a prodrug. The EC_50_ value of this ester was then evaluated on PC3 cells, and a value of (2.3±0.8) μm was obtained.

## Conclusions

In conclusion, we report herein the discovery of 3‐{[4‐(2‐morpholino‐4‐oxo‐1,4‐dihydroquinolin‐8‐yl)‐1*H*‐1,2,3‐triazol‐1‐yl]methyl}benzoic acid (**37**), a novel, potent pan‐phosphoinositide 3‐kinase (PI3K) inhibitor that bears a strategic carboxylic acid, and the design of its ester prodrugs. Esterification of **37** generated molecules that were inactive in the enzymatic assay but that could cross cell membranes, and after ester hydrolysis, they inhibited the PI3Ks. To date, few prodrug PI3K inhibitors have been reported. One is a prostate‐cancer‐specific PI3K inhibitor generated by coupling a chemically modified form of the quercetin analogue LY294002 with the peptide Mu‐LEHSSKLQL, in which the internal sequence HSSKLQ is a substrate for the prostate‐specific antigen protease.^23^ The second prodrug was derived by conjugation of LY294002 to the amino terminus of the Arg‐Gly‐Asp‐Ser integrin binding tetrapeptide through a morpholinium methylsuccinate.^24^ However, in both prodrugs the biologically active portion was able to cross cell membranes, which raises questions on side effects mediated by uncontrolled systemic inhibition of PI3Ks. Recently, Abbott et al. reported the generation of tetrazole analogues of LY294002 active on the α isoform of PI3K only if delivered as a prodrug.^25^ Although different cancer cell lines were used, an EC_50_ value comparable with the one displayed by our compound, methyl 3‐{[4‐(2‐morpholino‐4‐oxo‐1,4‐dihydroquinolin‐8‐yl)‐1*H*‐1,2,3‐triazol‐1‐yl]methyl}benzoate (**64**), was reported [EC_50_=(2.1±1.1) μm on MCF‐7 versus (2.3±0.8) μm on PC3].

Although pan‐PI3K inhibitors are emerging as a new opportunity in the treatment of non‐cancer‐related diseases, data derived from clinical trials has demonstrated that treatment with pan‐PI3K inhibitors causes several side effects, including nausea, diarrhea, rash, and increased released of insulin in plasma. Therefore, the unique properties of the prodrugs described in this manuscript might be considered of great relevance in the context of systemic toxicity management. Indeed, these derivatives might explicate their activity exclusively within the cell, after conversion into **37**, which might in turn decrease systemic toxicity. Their short half‐lives, both in microsomes and plasma, highlight the therapeutic potential in topical administration (e.g., aerosol therapy or epicutaneous treatment), which might overcome toxicity and avoid systemic exposure. Indeed, several pieces of evidence suggest the use of pan‐PI3K inhibitors in topical administration.^26^, ^27^ Further studies are underway and will be performed to demonstrate the efficacy of compound **64** in different mouse models of chronic inflammation, for which topical administration would be beneficial, such as asthma and psoriasis, and to evaluate its systemic toxicity.

## Experimental Section

### General procedures

Commercially available reagents and solvents were purchased from Aldrich or Alfa Aesar and were used without further purification. Tetrahydrofuran (THF) and toluene were distilled immediately before use from Na/benzophenone under a slight positive atmosphere of dry nitrogen. Ethylenediamine was distilled immediately before use from CaH_2_ under a slight positive atmosphere of dry nitrogen. *N*,*N*′‐Dimethylformamide (DMF) was distilled under vacuum from KOH and was stored on activated molecular sieves (4 Å). Dichloromethane was dried by distillation from P_2_O_5_ and was stored on activated molecular sieves (4 Å). If needed, the reactions were performed in flame‐ or oven‐dried glassware under a positive pressure of dry nitrogen. Melting points were determined in open glass capillaries with a Stuart scientific SMP3 apparatus. Compounds were checked by IR (FTIR THERMO‐NICOLET AVATAR), ^1^H NMR and ^13^C NMR APT (JEOL ECP 300 MHz spectrometer), and mass spectrometry (Thermo Finnigan LCQ‐deca XP‐plus) equipped with an ESI source and an ion‐trap detector. Chemical shifts are reported in parts per million (ppm). Flash column chromatography was performed on silica gel (Kieselgel 60, 230–400 mesh ASTM). Thin‐layer chromatography (TLC) was performed on 5×20 cm plates with a layer thickness of 0.25 mm (Merck Silica gel 60 F_254_). If necessary, they were developed with KMnO_4_ reagent. The purity of the tested compounds was established by elemental analyses. Elemental analyses (C, H, N) of the compounds that underwent biological evaluation were within ±0.4 % of the calculated values, confirming ≥95 % purity.

### Synthesis

**5‐[Bis(Methylthio)methylene]‐2,2‐dimethyl‐1,3‐dioxane‐4,6‐dione (16)**: TEA (48.0 mL, 0.346 mmol) and CS_2_ (10.2 mL, 0.17 mmol) were added to a solution of Meldrum's acid (25.0 g, 0.17 mmol) in DMSO (300 mL). The mixture was stirred for 3 h at room temperature, and then iodomethane (2 equiv) was slowly added at 0 °C. The mixture was stirred overnight at room temperature. Ice was added to the flask to promote precipitation of the product, which was then filtered to yield compound **16** as a yellow solid (34.1 g, 81 %).

**5‐[(2‐Iodophenylamino)(methylthio)methylene]‐2,2‐dimethyl‐1,3‐dioxane‐4,6‐dione (18)**: A mixture of 5‐[bis(methylthio)methylene]‐2,2‐dimethyl‐1,3‐dioxane‐4,6‐dione (453 mg, 1.83 mmol) and 2‐iodoaniline (400 mg, 1.83 mmol) in ethanol (4 mL) was heated at 80 °C for 4 h. During the reaction, the product precipitated. Filtration yielded **18** as a yellow solid (522 mg, 68 %).

**5‐[(2‐Iodophenylamino)(morpholino)methylene]‐2,2‐dimethyl‐1,3‐dioxane‐4,6‐dione (20)**: A mixture of 5‐[(2‐iodophenylamino)(methylthio) methylene]‐2,2‐dimethyl‐1,3‐dioxane‐4,6‐dione (500 mg, 1.23 mmol) and morpholine (214 μL, 2.46 mmol) in THF (5 mL) was heated at reflux overnight. The volatiles were evaporated under reduced pressure. Diethyl ether (10 mL) was added, and the solid was filtered and washed with EtOAc (10 mL) to give **20** as a white powder (429 mg, 76 %).

**8‐Iodo‐2‐morpholinoquinolin‐4(1*H*)‐one (21)**: 5‐[(2‐Iodophenylamino)(morpholino)methylene]‐2,2‐dimethyl‐1,3‐dioxane‐4,6‐dione (1 g, 2.18 mmol) was heated in diphenyl ether (10 mL) at reflux for 30 min. The crude brown oil, after purification by column chromatography (petroleum ether/EtOAc 5:5 to 0:1), yielded **21** as a white powder (519 mg, 67 %).

**2‐Morpholino‐8‐[(trimethylsilyl)ethynyl] quinolin‐4(1*H*)‐one (22)**: Under a nitrogen atmosphere, PdCl_2_(PPh_3_)_2_ (6 mg, 0.00843 mmol), Cu^I^ (5 mg, 0.0252 mmol), DIPEA (48 μL, 0.280 mmol), and trimethylsilylacetylene (39 μL, 0.280 mmol) were added to a solution of 8‐iodo‐2‐morpholinoquinolin‐4(1*H*)‐one (100 mg, 0.280 mmol) in toluene (2 mL) and DMF (two drops). The mixture was stirred at room temperature for 2 h. The mixture was diluted with water (10 mL) and extracted with EtOAc (5×10 mL). The combined organic layer was dried with sodium sulfate. The crude material was purified by column chromatography (EtOAc/MeOH 1:0 to 95:5) to yield **22** as a yellow solid (66 mg, 72 %).

**8‐Ethynyl‐2‐morpholinoquinolin‐4(1*H*)‐one (12)**: Potassium carbonate (949 mg, 6.88 mmol) was added to a solution of 2‐morpholino‐8‐[(trimethylsilyl)ethynyl]quinolin‐4(1*H*)‐one (1.87 g, 5.74 mmol) in methanol (20 mL). The mixture was stirred at room temperature for 3 h. The volatiles were removed in vacuo. The mixture was diluted with brine (20 mL) and extracted with CH_2_Cl_2_ (4×10 mL). The combined organic layer was dried with sodium sulfate. The crude material was purified by column chromatography (EtOAc/MeOH 1:0 to 9:1) to yield **12** as a yellow solid (1.23 g, 84 %).

**4‐Morpholino‐4*H*‐pyrrolo[3,2,1‐*ij*]quinolin‐6(5*H*)‐one (24)**: Alkyne **12** (78 mg, 0.31 mmol) was added to a suspension of phenyl azide (**23**; 100 mg, 0.31 mmol) in water (570 μL) and *t*BuOH (570 μL). Then, 1 m aqueous sodium ascorbate (29 μL) and copper sulfate pentahydrate (0.72 mg, 0.29 mmol) were added under vigorous stirring. The product precipitated, and it was filtered and washed with water (2×5 mL) and diethyl ether (2×5 mL) to give **24** as a yellow solid (49 mg, 62 %).

**General procedure for the synthesis of compounds 25**–**54**: TBTA (0.0040 mmol, 0.05 equiv) was added to a solution of Cu(OAc)_2_ (0.0040 mmol, 0.05 equiv) in THF (0.3 mL), and the resulting mixture was stirred at room temperature for 30 min. A solution of azide **13** (0.079 mmol, 1 equiv) in THF (0.3 mL), a solution of alkyne **12** (0.079 mmol, 1 equiv) in THF (0.3 mL), and a solution of sodium ascorbate (0.0079 mmol, 0.1 equiv) in a minimum amount of water were added. The mixture was stirred at room temperature overnight. The product precipitated, and it was filtered and washed with water (2×5 mL) and diethyl ether (2×5 mL) to give a solid. The crude product was then screened, and compounds **25**, **35**, **37**, **45**, **48**, and **54** were resynthesized and subjected to column chromatography (EtOAc and EtOAc/MeOH 9:1).

**2‐Morpholino‐8‐(1‐phenyl‐1*H*‐1,2,3‐triazol‐4‐yl)quinolin‐4(1*H*)‐one (25)**: Yellow solid (90 %): mp: 244–245 °C (dec.); ^1^H NMR (300 MHz, [D_6_]DMSO): *δ*=9.30 (s, 1 H), 8.39 (br s, 1 H), 7.96–7.91 (m, 4 H), 7.63 (t, *J*=7.7 Hz, 2 H), 7.49 (t, *J*=7.7 Hz, 1 H), 7.30 (t, *J*=7.7 Hz, 1 H), 6.51 (s, 1 H), 3.79–3.73 (m, 4 H), 3.59–3.53 ppm (m, 4 H); ^13^C NMR (75 MHz, [D_6_]DMSO): *δ*=160.7, 152.8, 137.3, 136.4, 131.1, 130.6 (2C), 129.2, 128.7, 126.3, 124.3, 121.9, 121.7, 120.6, 91.8, 66.5, 46.4 ppm; IR (KBr): $\tilde{\nu }$ =$\tilde{\nu }$ 3437, 3122, 1636, 1584, 1500, 1237, 1124, 795, 761, 688 cm^−1^; MS (ESI): *m*/*z*=374 [*M*+H]^+^; elemental analysis calcd (%) for C_21_H_19_N_5_O_2_ (373.41): C 67.55, H 5.13, N 18.76; found: C 67.71, H 5.25, N 18.60.

**8‐[1‐(3‐Hydroxyphenyl)‐1*H*‐1,2,3‐triazol‐4‐yl]‐2‐morpholinoquinolin‐4(1*H*)‐one (26)**: Yellow solid (90 %).

**8‐[1‐(3‐Hydroxy‐4‐methoxyphenyl)‐1*H*‐1,2,3‐triazol‐4‐yl]‐2‐morpholinoquinolin‐4(1*H*)‐one (27)**: Yellow solid (40 %).

**8‐[1‐(4‐Chlorophenyl)‐1*H*‐1,2,3‐triazol‐4‐yl]‐2‐morpholinoquinolin‐4(1*H*)‐one (28)**: Gray solid (50 %).

**2‐Morpholino‐8‐{1‐[4‐(trifluoromethoxy)phenyl]‐1*H*‐1,2,3‐triazol‐4‐yl}quinolin‐4(1*H*)‐one (29)**: Yellow solid (69 %).

**8‐[1‐(4‐Methoxy‐3‐nitrophenyl)‐1*H*‐1,2,3‐triazol‐4‐yl]‐2‐morpholinoquinolin‐4(1*H*)‐one (30)**: Yellow solid (50 %).

**Methyl‐4‐{[4‐(2‐morpholino‐4‐oxo‐1,4‐dihydroquinolin‐8‐yl)‐1*H*‐1,2,3‐triazol‐1‐yl]methyl}benzoate (31)**: Gray solid (90 %).

**8‐[1‐(4‐Aminophenyl)‐1*H*‐1,2,3‐triazol‐4‐yl]‐2‐morpholinoquinolin‐4(1*H*)‐one (32)**: Brown solid (65 %).

**2‐{4‐[4‐(2‐Morpholino‐4‐oxo‐1,4‐dihydroquinolin‐8‐yl)‐1*H*‐1,2,3‐triazol‐1‐yl]phenyl} acetonitrile (33)**: Yellow solid (74 %).

**2‐Morpholino‐8‐[1‐(naphthalen‐2‐yl)‐1*H*‐1,2,3‐triazol‐4‐yl]quinolin‐4(1*H*)‐one (34)**: Yellow solid (68 %).

**8‐[1‐(3‐Methoxyphenyl)‐1*H*‐1,2,3‐triazol‐4‐yl]‐2‐morpholinoquinolin‐4(1*H*)‐one (35)**: Yellow solid (57 %): mp: 141–142 °C (dec.); ^1^H NMR (300 MHz, CD_3_OD): *δ*=9.03 (s, 1 H), 8.08 (d, *J*=7.7 Hz, 1 H), 7.92 (d, *J*=7.7 Hz, 1 H), 7.47–7.45 (m, 3 H), 7.26 (t, *J*=7.7 Hz, 1 H), 7.08–7.04 (m, 1 H), 5.79 (s, 1 H), 3.89 (s, 3 H), 3.84 (t, *J*=4.9 Hz, 4 H), 3.49 ppm (t, *J*=4.9 Hz, 4 H); ^13^C NMR (75 MHz, [D_6_]DMSO): *δ*=160.8, 138.4, 134.5, 133.8, 132.6 (2 C), 131.5, 128.7, 123.1, 121.6, 121.2, 115.2, 113.6, 112.5, 110.9, 106.0, 92.1, 66.5, 56.1, 45.7 ppm; IR (KBr): $\tilde{\nu }$ =3263, 2965, 1622, 1583, 1495, 1235, 1123, 1003, 871, 791 cm^−1^; MS (ESI): *m*/*z*=404 [*M*+H]^+^; elemental analysis calcd (%) for C_22_H_21_N_5_O_3_ (403.43): C 65.50, H 5.25, N 17.36; found: C 65.22, H 5.27, N 17.33.

**2‐Morpholino‐8‐[1‐(quinolin‐3‐yl)‐1*H*‐1,2,3‐triazol‐4‐yl]quinolin‐4(1*H*)‐one (36)**: Yellow solid (93 %).

**3‐{[4‐(2‐Morpholino‐4‐oxo‐1,4‐dihydroquinolin‐8‐yl)‐1*H*‐1,2,3‐triazol‐1‐yl]methyl}benzoic acid (37)**: Yellow solid (70 %): mp: 290–291 °C (dec.); ^1^H NMR (300 MHz, [D_6_]DMSO): *δ*=8.08 (s, 1 H), 8.00 (d, *J*=7.7 Hz, 1 H), 7.93 (d, *J*=3.3 Hz, 1 H), 7.88 (d, *J*=7.7 Hz, 1 H), 7.51 (t, *J*=7.7 Hz, 1 H), 7.34–7.29 (m, 2 H), 7.02 (d, *J*=3.3 Hz, 1 H), 6.50 (s, 1 H), 5.75 (s, 2 H), 3.85–3.81 (m, 4 H), 3.26–3.24 ppm (m, 4 H); ^13^C NMR (75 MHz, [D_6_]DMSO): *δ*=162.4, 159.6, 145.1, 136.2, 134.5, 133.6, 133.1, 130.2, 130.0, 128.2, 126.0, 125.6, 125.2, 122.3, 121.5, 121.2, 118.3, 91.9, 66.5, 53.2, 45.8 ppm; IR (KBr): $\tilde{\nu }$ =2970, 2866, 1707, 1636, 1582, 1444, 1242, 1117, 789, 739 cm^−1^; MS (ESI): *m*/*z*=432 [*M*+H]^+^; elemental analysis calcd (%) for C_23_H_21_N_5_O_4_ (431.44): C 64.03, H 4.91, N 16.23; found: C 64.00, H 4.98, N 16.20.

***N*** **‐{4‐Methoxy‐3‐[4‐(2‐morpholino‐4‐oxo‐1,4‐dihydroquinolin‐8‐yl)‐1*H*‐1,2,3‐triazol‐1‐yl]phenyl}acetamide (38)**: Yellow solid (78 %).

**8‐{1‐[3‐Methoxy‐5‐(trifluoromethoxy)phenyl]‐1*H*‐1,2,3‐triazol‐4‐yl}‐2‐morpholinoquinolin‐4(1*H*)‐one (39)**: Yellow solid (90 %).

**8‐[1‐(4‐Hydroxy‐2‐methylphenyl)‐1*H*‐1,2,3‐triazol‐4‐yl]‐2‐morpholinoquinolin‐4(1*H*)‐one (40)**: Brown solid (31 %).

**8‐{1‐[2‐(Hydroxymethyl)phenyl]‐1*H*‐1,2,3‐triazol‐4‐yl}‐2‐morpholinoquinolin‐4(1*H*)‐one (41)**: Yellow solid (40 %).

**Methyl 4‐[4‐(2‐morpholino‐4‐oxo‐1,4‐dihydroquinolin‐8‐yl)‐1*H*‐1,2,3‐triazol‐1‐yl]benzoate (42)**: Yellow solid (90 %).

**8‐[1‐(3,4‐Dimethoxyphenyl)‐1*H*‐1,2,3‐triazol‐4‐yl]‐2‐morpholinoquinolin‐4(1*H*)‐one (43)**: Gray amorphous solid (76 %).

**8‐[1‐(4‐Methoxyphenyl)‐1*H*‐1,2,3‐triazol‐4‐yl]‐2‐morpholinoquinolin‐4(1*H*)‐one (44)**: Light‐yellow solid (55 %).

**4‐[4‐(2‐Morpholino‐4‐oxo‐1,4‐dihydroquinolin‐8‐yl)‐1*H*‐1,2,3‐triazol‐1‐yl]benzoic acid (45)**: Yellow solid (66 %): mp: 269–270 °C (dec.); ^1^H NMR (300 MHz, [D_6_]DMSO): *δ*=9.39 (s, 1 H), 8.42 (d, *J*=6.9 Hz, 2 H), 8.11–8.09 (m, 2 H), 7.97 (d, *J*=6.9 Hz, 2 H), 7.33 (t, *J*=8.2 Hz, 1 H), 6.54 (s, 1 H), 3.79–3.77 (m, 4 H), 3.60–3.58 ppm (m, 4 H); ^13^C NMR (75 MHz, [D_6_]DMSO): *δ*=161.3, 159.8, 138.2, 136.2, 133.3, 132.6, 131.7, 131.4, 128.7, 126.4, 124.1, 123.8, 121.2, 120.6, 111.8, 92.0, 66.3, 46.3 ppm; IR (KBr): $\tilde{\nu }$ =3336, 3128, 1697, 1620, 1585, 1414, 1238, 811, 768, 750 cm^−1^; MS (ESI): *m*/*z*=418 [*M*+H]^+^; elemental analysis calcd (%) for C_22_H_19_N_5_O_4_ (417.42): C 63.30, H 4.59, N 16.78; found: C 63.62, H 4.95, N 16.48.

**8‐[1‐(Benzo[*d*][1,3]dioxol‐5‐yl)‐1*H*‐1,2,3‐triazol‐4‐yl]‐2‐morpholinoquinolin‐4(1*H*)‐one (46)**: Yellow solid (91 %).

**8‐[1‐(3,5‐Dimethoxyphenyl)‐1*H*‐1,2,3‐triazol‐4‐yl]‐2‐morpholinoquinolin‐4(1*H*)‐one (47)**: Yellow solid (84 %).

**8‐(1‐Benzyl‐1*H*‐1,2,3‐triazol‐4‐yl)‐2‐morpholinoquinolin‐4(1*H*)‐one (48)**: Light‐yellow solid (84 %): mp: 90–91 °C; ^1^H NMR (300 MHz, CD_3_OD): *δ*=8.57 (s, 1 H), 8.03 (d, *J*=7.7 Hz, 1 H), 7.83 (d, *J*=7.7 Hz, 1 H), 7.42–7.38 (m, 5 H), 7.24 (t, *J*=7.7 Hz, 1 H), 5.81 (s, 1 H), 5.68 (s, 2 H), 3.88–3.85 (m, 4 H), 3.53–3.50 ppm (m, 4 H); ^13^C NMR (75 MHz, [D_6_]DMSO): *δ*=160.2, 145.2, 136.5, 131.8, 130.4, 129.4, 129.0, 128.2, 127.4, 127.3, 125.1, 122.3, 121.6, 118.6, 91.9, 66.5, 53.6, 45.8 ppm; IR (KBr): $\tilde{\nu }$ =3264, 3142, 1628, 1586, 1501, 1445, 1255, 1117, 785, 728 cm^−1^; MS (ESI): *m*/*z*=388 [*M*+H]^+^; elemental analysis calcd (%) for C_22_H_21_N_5_O_2_ (387.43): C 68.20, H 5.46, N 18.08; found: C 68.18, H 5.72, N 17.83.

**Methyl 3‐[4‐(2‐morpholino‐4‐oxo‐1,4‐dihydroquinolin‐8‐yl)‐1*H*‐1,2,3‐triazol‐1‐yl]benzoate (49)**: Yellow solid (62 %).

**4‐[4‐(2‐Morpholino‐4‐oxo‐1,4‐dihydroquinolin‐8‐yl)‐1*H*‐1,2,3‐triazol‐1‐yl]benzenesulfonamide (50)**: Yellow solid (95 %).

**8‐[1‐(2‐Methoxyphenyl)‐1*H*‐1,2,3‐triazol‐4‐yl]‐2‐morpholinoquinolin‐4(1*H*)‐one (51)**: Yellow amorphous solid (90 %).

**3‐[4‐(2‐Morpholino‐4‐oxo‐1,4‐dihydroquinolin‐8‐yl)‐1*H*‐1,2,3‐triazol‐1‐yl]benzoic acid (52)**: Yellow solid (89 %).

**2‐Morpholino‐8‐[1‐(naphthalen‐1‐yl)‐1*H*‐1,2,3‐triazol‐4‐yl]quinolin‐4(1*H*)‐one (53)**: Yellow solid (90 %).

**8‐[1‐(2‐Hydroxyphenyl)‐1*H*‐1,2,3‐triazol‐4‐yl]‐2‐morpholinoquinolin‐4(1*H*)‐one (54)**: Brown solid (77 %): mp: 253–254 °C (dec.); ^1^H NMR (300 MHz, [D_6_]DMSO): *δ*=9.38 (s, 1 H), 8.43 (br s, 1 H), 7.94 (d, *J*=7.7 Hz, 1 H), 7.78 (d, *J*=7.1 Hz, 1 H), 7.31–7.26 (m, 3 H), 7.15 (d, *J*=7.7 Hz, 1 H), 7.00 (t, *J*=7.7 Hz, 1 H), 6.55 (s, 1 H), 3.76–3.74 (m, 4 H), 3.56–3.54 ppm (m, 4 H); ^13^C NMR (75 MHz, [D_6_]DMSO): *δ*=159.5, 154.8, 149.4, 145.2, 141.3, 130.1, 128.3, 127.0, 126.0, 125.2, 124.7, 121.6, 120.3, 117.9, 109.9, 101.3, 92.0, 66.6, 51.9 ppm; IR (KBr): $\tilde{\nu }$ =3230, 2949, 1632, 1586, 1236, 1123, 800, 750 cm^−1^; MS (ESI): *m*/*z*=390 [*M*+H]^+^; elemental analysis calcd (%) for C_21_H_19_N_5_O_3_ (389.41): C 64.77, H 4.92, N 17.98; found: C 64.88, H 5.17, N 18.21.

**4‐[4‐(2‐Morpholino‐4‐oxo‐1,4‐dihydroquinolin‐8‐yl)‐1*H*‐1,2,3‐triazol‐1‐yl]butanoic acid (55)**: Yellow solid (47 %).

**5‐[4‐(2‐Morpholino‐4‐oxo‐1,4‐dihydroquinolin‐8‐yl)‐1*H*‐1,2,3‐triazol‐1‐yl]pentanoic acid (56)**: Yellow solid (40 %).

**6‐84‐(2‐Morpholino‐4‐oxo‐1,4‐dihydroquinolin‐8‐yl)‐1*H*‐1,2,3‐triazol‐1‐yl]hexanoic acid (57)**: Yellow solid (48 %).

***N*** **‐(Methylsulfonyl)‐3‐{[4‐(2‐morpholino‐4‐oxo‐1,4‐dihydroquinolin‐8‐yl)‐1*H*‐1,2,3‐triazol‐1‐yl]methyl}benzamide (58)**: Yellow solid (69 %).

**3‐{[4‐(2‐Morpholino‐4‐oxo‐1,4‐dihydroquinolin‐8‐yl)‐1*H*‐1,2,3‐triazol‐1‐yl]methyl}benzamide (59)**: Yellow solid (95 %).

***N*** **‐Benzyl‐3‐{[4‐(2‐morpholino‐4‐oxo‐1,4‐dihydroquinolin‐8‐yl)‐1*H*‐1,2,3‐triazol‐1‐yl]methyl}benzamide (60)**: Yellow solid (24 %).

***N*** **‐Cyclopropyl‐3‐{[4‐(2‐morpholino‐4‐oxo‐1,4‐dihydroquinolin‐8‐yl)‐1*H*‐1,2,3‐triazol‐1‐yl]methyl}benzamide (61)**: Yellow solid (31 %).

***N***,***N*** **‐Diethyl‐3‐{[4‐(2‐morpholino‐4‐oxo‐1,4‐dihydroquinolin‐8‐yl)‐1*H*‐1,2,3‐triazol‐1‐yl]methyl}benzamide (62)**: Yellow solid (40 %).

**8‐{1‐[3‐(1*H*‐Tetrazol‐5‐yl)benzyl]‐1*H*‐1,2,3‐triazol‐4‐yl}‐2‐morpholinoquinolin‐4(1*H*)‐one (63)**: 3‐{[4‐(2‐Morpholino‐4‐oxo‐1,4‐dihydroquinolin‐8‐yl)‐1*H*‐1,2,3‐triazol‐1‐yl]methyl}benzonitrile was prepared following the general procedure for the click reaction. Yellow solid (95 %). ^1^H NMR (300 MHz, [D_6_]DMSO): *δ*=8.75 (s, 1 H), 8.36 (d, *J*=7.1 Hz, 1 H), 7.92 (s, 1 H), 7.86 (m, 2 H), 7.74 (d, *J*=7.1 Hz, 1 H), 7.63 (t, *J*=7.1 Hz, 1 H), 7.28 (t, *J*=7.3 Hz, 1 H), 6.49 (s, 1 H), 5.80 (s, 2 H), 3.74–3.70 (m, 4 H), 3.47–3.41 ppm (m, 4 H); MS (ESI): *m*/*z*=413 [*M*+H]^+^.

Sodium azide (46.8 mg, 0.72 mmol) was added to a solution of 3‐{[4‐(2‐morpholino‐4‐oxo‐1,4‐dihydroquinolin‐8‐yl)‐1*H*‐1,2,3‐triazol‐1‐yl]methyl}benzonitrile (200.0 mg, 0.48 mmol) in DMF (1.2 mL) and methanol (1.2 mL). The mixture was stirred at 110 °C overnight. Diethyl ether (3 mL) was added to the mixture, and the product precipitated. It was filtered to yield **63** as a brown solid (214 mg, 98 %).

**General procedure for the synthesis of ester prodrugs 64–76**: TBTA (0.0040 mmol, 0.05 equiv) was added to a solution of Cu(OAc)_2_ (0.0040 mmol, 0.05 equiv) in THF (0.3 mL), and the resulting mixture was stirred at room temperature for 30 min. A solution of the azide (0.079 mmol, 1 equiv) in THF (0.3 mL), a solution of alkyne **12** (0.079 mmol, 1 equiv) in THF (0.3 mL), and a solution of sodium ascorbate (0.0079 mmol, 0.1 equiv) in a minimum amount of water were added. The mixture was stirred at room temperature overnight. The product precipitated, and it was filtered and washed with water (2×5 mL) and diethyl ether (2×5 mL) to give a solid. The crude product was subjected to column chromatography (EtOAc and EtOAc/MeOH 9:1).

**Methyl 3‐{[4‐(2‐morpholino‐4‐oxo‐1,4‐dihydroquinolin‐8‐yl)‐1*H*‐1,2,3‐triazol‐1‐yl]methyl}benzoate (64)**: White solid (95 %): mp: 185.5–186.5 °C; ^1^H NMR (300 MHz, [D_6_]DMSO): *δ*=8.72 (s, 1 H), 8.37 (d, *J*=7.7 Hz, 1 H), 8.02 (s, 1 H), 7.97–7.90 (m, 2 H), 7.71 (d, *J*=7.7 Hz, 1 H), 7.59 (t, *J*=7.7 Hz, 1 H), 7.28 (t, *J*=7.9 Hz, 1 H), 6.47 (s, 1 H), 5.82 (s, 2 H), 3.85 (s, 3 H), 3.74–3.63 (m, 4 H), 3.58–3.24 ppm (m, 4 H); ^13^C NMR (75 MHz, CDCl_3_): *δ*=166.3, 147.7, 134.4, 132.7, 131.5, 130.4, 129.8, 129.7, 129.5, 128.7, 126.4, 123.3, 122.4, 120.8, 118.9, 91.9, 66.3, 54.4, 52.5, 46.3 ppm; IR (KBr): $\tilde{\nu }$ =3422, 1720, 1582, 1287, 1130, 732 cm^−1^; MS (ESI): *m*/*z*=446 [*M*+H]^+^; elemental analysis calcd (%) for C_24_H_23_N_5_O_4_ (445.47): C 64.71, H 5.20, N 15.72; found: C 64.31, H 5.47, N 15.70.

**Benzyl 3‐{[4‐(2‐morpholino‐4‐oxo‐1,4‐dihydroquinolin‐8‐yl)‐1*H*‐1,2,3‐triazol‐1‐yl]methyl}benzoate (65)**: White solid (99 %).

**Isopropyl 3‐{[4‐(2‐morpholino‐4‐oxo‐1,4‐dihydroquinolin‐8‐yl)‐1*H*‐1,2,3‐triazol‐1‐yl]methyl}benzoate (66)**: Yellow solid (52 %).

**Ethyl 3‐{[4‐(2‐morpholino‐4‐oxo‐1,4‐dihydroquinolin‐8‐yl)‐1*H*‐1,2,3‐triazol‐1‐yl]methyl}benzoate (67)**: White solid (64 %).

**Butyl 3‐{[4‐(2‐morpholino‐4‐oxo‐1,4‐dihydroquinolin‐8‐yl)‐1*H*‐1,2,3‐triazol‐1‐yl]methyl}benzoate (68)**: Yellow solid (66 %).

***tert*** **‐Butyl 3‐{[4‐(2‐morpholino‐4‐oxo‐1,4‐dihydroquinolin‐8‐yl)‐1*H*‐1,2,3‐triazol‐1‐yl]methyl}benzoate (69)**: Yellow solid (28 %).

**Pyridin‐4‐ylmethyl 3‐{[4‐(2‐morpholino‐4‐oxo‐1,4‐dihydroquinolin‐8‐yl)‐1*H*‐1,2,3‐triazol‐1‐yl]methyl}benzoate (70)**: Yellow solid (79 %).

**2‐Morpholinoethyl 3‐{[4‐(2‐morpholino‐4‐oxo‐1,4‐dihydroquinolin‐8‐yl)‐1*H*‐1,2,3‐triazol‐1‐yl]methyl}benzoate (71)**: Yellow solid (45 %).

**Benzo[*d*][1,3]dioxol‐5‐yl methyl 3‐{[4‐(2‐morpholino‐4‐oxo‐1,4‐dihydroquinolin‐8‐yl)‐1*H*‐1,2,3‐triazol‐1‐yl]methyl}benzoate (72)**: Yellow solid (72 %).

**Isopentyl 3‐{[4‐(2‐morpholino‐4‐oxo‐1,4‐dihydroquinolin‐8‐yl)‐1*H*‐1,2,3‐triazol‐1‐yl]methyl}benzoate (73)**: Yellow solid (78 %).

**Pentan‐2‐yl 3‐{[4‐(2‐morpholino‐4‐oxo‐1,4‐dihydroquinolin‐8‐yl)‐1*H*‐1,2,3‐triazol‐1‐yl]methyl}benzoate (74)**: Yellow solid (63 %).

**Undecyl 3‐{[4‐(2‐morpholino‐4‐oxo‐1,4‐dihydroquinolin‐8‐yl)‐1*H*‐1,2,3‐triazol‐1‐yl]methyl}benzoate (75)**: White solid (58 %).

**Octadecyl 3‐{[4‐(2‐morpholino‐4‐oxo‐1,4‐dihydroquinolin‐8‐yl)‐1*H*‐1,2,3‐triazol‐1‐yl]methyl}benzoate (76)**: Yellow solid (84 %).

### Biological methods

*In vitro selection, lipid kinase assay*: To determine the inhibitory activity of the new molecular entities on PI3K enzyme, a lipid kinase assay was performed: recombinant proteins (30 ng), human PI3Kα (PIK3CA/PIK3R1, #1161‐1165‐1 ProQinase–Germany), hPI3Kβ (PIK3CB wt/PIK3R1, #1168‐1165‐1 ProQinase, Germany), hPI3Kγ (PIK3CG, #1163‐0000‐1 ProQinase, Germany), hPI3Kδ (PIK3CD/PIK3R1, #1162–1165‐1 ProQinase, Germany), hPI3K‐C2α (PIK3C2A, #14‐906 Merck Millipore_Merck KGaA, Germany), hPI3K‐C2β (PIK3C2B, #14‐907 Merck Millipore_Merck KGaA, Germany), hPI3K‐c2γ (PIK3C2G, #1207‐0000‐1 ProQinase, Germany), and hVps34 (PIK3C3, #1160‐0000‐1 ProQinase, Germany), were incubated in kinase buffer {12.5 μL; 20 mm Tris‐HCl pH 7.5, 10 mm MgCl_2_, 10 μm ATP, 0.05 mg mL^−1^ BSA, 0.5 mm EDTA, 5 mm HEPES pH 7.5, for PI3Kα,PI3Kβ, PI3Kγ, PI3Kδ, PI3KC‐2α, and PI3KC‐2γ; 15 mm MnCl_2_, 5 mm EGTA, 100 μm ATP, 50 mm HEPES pH 7.5, 100 mm NaCl, and 0.03 % CHAPS for PI3K‐C2β and VPS34 {BSA=bovine serum albumin; EDTA=ethylenediaminetetraacetic acid; HEPES=4‐(2‐hydroxyethyl)‐1‐piperazineethanesulfonic acid; EGTA=ethylene glycol‐bis(2‐aminoethylether)‐*N*,*N*,*N*′,*N*′‐tetraacetic acid; CHAPS=3‐[(3‐cholamidopropyl)dimethylammonio]‐1‐propanesulfonate}. In the reaction, the ATP concentration used for different PI3Ks was defined on the basis of the Michaelis–Menten kinetics of each enzyme, provided by the supplier. Reaction was performed in presence of 0.5 mg mL^−1^ lipid micelles (1:1 phosphatidylinositol/phosphatidylserine) and in the absence (DMSO alone) or presence of increasing concentrations of each compound (from 0.1 nm to 30 μm). The mixture was kept at room temperature (RT) for 30 min for PI3Kα, PI3Kβ, PI3Kγ, PI3Kδ, PI3KC‐2α, and PI3KC‐2γ and for 1 h for PI3K‐C2β and PI3K‐C3. The amount of ADP produced in the reaction was measured by using the ADP‐Glo Kinase Assay kit from Promega (#V9102) and a luminescence reader (Glomax multi detection system, Promega, model number 9301‐010).

The residual PI3K lipid kinase activity in the presence of each molecule at different concentrations was related to the control, intended as the recombinant protein in the presence of DMSO alone and was expressed as a percentage. To derive the IC_50_ value, all data (% of lipid kinase activity) were plotted on a dose–response curve (Graph Pad software), and the IC_50_ was calculated by using nonlinear regression fit (equation [log agonist] versus response).

*Lineweaver–Burk plot*: The linear phase of the kinetic reaction was defined with 2.4 μg mL^−1^ of PI3Kδ in a 20 min reaction time. In the presence of increasing concentrations of **37** (5, 10 and 20 nm), the PI3K lipid kinase activity (for the assay method see “In vitro selection, lipid kinase assay” in the Experimental Section) was assayed at various concentrations of ATP (5, 10, 25, 50, and 100 μm) and in the presence of 0.5 mg mL^−1^ lipid micelles (1:1 phosphatidylinositol/phosphatidylserine). A Lineweaver–Burk plot was obtained by plotting 1/*v*, for which *v* represents pmol min^−1^ of ADP produced in the reaction measured with the ADP‐Glo Kinase Assay kit from Promega (#V9102) versus 1/[ATP] (the inverse of the ATP concentration). The minus‐enzyme control was obtained by incubating phosphatidylinositol in the absence of PI3Kδ.

*Western blot analysis*: PC3 cells were seeded in 96‐well plates in the presence of free Dulbecco's modified Eagle's medium (DMEM) high‐glucose GlutaMAX^TM^ (Gibco), 10 % fetal bovine serum (Invitrogen) supplemented with 5000 U mL^−1^ penicillin–streptomycin (Gibco). Subsequently, PC3 cells were incubated with increasing concentrations of **68** for 12 h. Cells were then lysed with a lysis buffer containing 20 mm Tris‐HCl pH 8, 138 mm NaCl, 2.7 mm KCl, 1 mm CaCl_2_, 5 mm EDTA pH 8, 1 mm MgCl_2_, 5 % glycerol, 1 % Triton X‐100, and protein extracts were analyzed by Western blot analysis. To assess the phosphorylation level of proteins involved in the PI3K/Akt signaling pathway, the following antibodies were used: phospho‐Akt (Ser473) (#4060 Cell Signaling Technology, USA), phospho‐Akt (Ser308) (#13038 Cell Signaling Technology, USA), Akt1 (#2967 Cell Signaling Technology, USA), phospho‐PRAS40 (Thr246) (#2640 Cell Signaling Technology, USA), PRAS40 (#2610 Cell Signaling Technology, USA), Phospho‐p70 S6 Kinase (Thr389) (Cell Signaling Technology #9205), Phospho‐GSK‐3α/β (Ser21/9) (Cell Signaling Technology #8566), GSK‐3α/β Cell Signaling Technology #5676), phospho‐FoxO1 (Thr24)/FoxO3a (Thr32) (Cell Signaling Technology #9464), FoxO3a (Cell Signaling Technology #2497), GAPDH (8C2) (Santa Cruz sc‐81545), and p70 S6 kinase α (H‐160) (Santa Cruz sc‐9027).

NIH3T3 cells were seeded in a 96‐well plate and starved overnight (ON) with serum‐free DMEM high glucose GlutaMAXTM (Gibco) supplemented with 5000 U mL^−1^ penicillin–streptomycin (Gibco). Cells were then incubated for 1 h with increasing concentrations of **64**, **37**, or DMSO. Subsequently, cells were stimulated for 5 min with 1 μm of insulin (#91077C, Sigma–Aldrich, USA). Cells were lysed and protein extracts were analyzed by western blot to assess the phosphorylation level of Akt on Ser473.

*Cell proliferation*: PC3 cell proliferation was measured with a 3‐(4,5‐dimethyl thiazol‐2‐yl)‐2,5‐diphenyltetrazolium bromide (MTT)‐based colorimetric assay (#11 465 007 001 Roche Diagnostic GmbH, Germany). PC3 cells were seeded in a 96‐well plate at a concentration of 10^4^ cells mL^−1^. PC3 cells were treated for 72 h with increasing concentrations of **64**, and MTT was added. After ON solubilization of the MTT metabolite, formazan, the absorbance (*λ*=550–600 nm absorbance, with a reference wavelength of 650 nm) of each well was read in a multi‐well reader (Glomax multi detection system, Promega, model number 9301‐010). The percentage of proliferating cells was calculated by relating each absolute value to the control intended as PC3 cells treated with DMSO alone. An IC_50_ value was then derived by plotting all data on a dose–response curve (Graph Pad software) by using nonlinear regression fit (equation [log agonist] versus response).

*Cell‐cycle analysis*: For DNA content determination, PC3 cells were seeded at 4×10^5^ cells per 6 cm dish in triplicate in the presence of DMEM high glucose GlutaMAXTM (Gibco), 10 % fetal bovine serum (Invitrogen) supplemented with 5000 U mL^−1^ penicillin–streptomycin (Gibco). The day after seeding, cells were treated for 48 h with 10 μm **64** or DMSO. Subsequently, PC3 cells were detached with 0.1 % trypsin–EDTA (Gibco), fixed in 70 % ethanol overnight at −20 °C, and stained for 40 min at 57 °C with PI solution, containing 0.05 % Triton X‐100, 0.1 mg mL^−1^ RNase, and 25 μg mL^−1^ propidium iodide. Sample were analyzed for the DNA content by using a FACS‐Calibur flow cytometer (Becton Dickinson Immunocytometry Systems, San Jose, USA). The percentages of cells in the G0/G1, S, and G2 phases were calculated.

*Analysis of apoptosis*: For Annexin V staining of apoptotic cells, PC3 cells were seeded at 4×10^5^ cells per 6 cm dish in triplicate. Cells were incubated 48 h in the presence or absence of 10 μm **64** and were then detached with 0.1 % trypsin–EDTA, resuspended in Annexin buffer (10 mm HEPES, pH 7.4, 140 mm NaCl, 2.5 mm CaCl_2_), and stained for Annexin V (FITC Annexin V #640906, BioLegend, USA). Samples were analyzed by using a FACS‐Calibur flow cytometer (Becton Dickinson Immunocytometry Systems, San Jose, USA).

Additional biological experiments, crystallographic details, the pdb file for the X‐ray crystal structure of 5NGB, and metabolism stability studies are available in the Supporting Information.

## Conflict of interest

*E.H. and G.C.T. are co‐founders of Kither Biotech S.r.l. The other authors declare no competing interests*.

## Supporting information

As a service to our authors and readers, this journal provides supporting information supplied by the authors. Such materials are peer reviewed and may be re‐organized for online delivery, but are not copy‐edited or typeset. Technical support issues arising from supporting information (other than missing files) should be addressed to the authors.

Supplementary
