# Supplementary material for: Identification of a Potent Phosphoinositide 3‐Kinase Pan Inhibitor Displaying a Strategic Carboxylic Acid Group and Development of Its Prodrugs
Source: ChemMedChem. 2017 Aug 31;12(18):1542–54. doi: 10.1002/cmdc.201700340 (PMC5697638; doi:10.1002/cmdc.201700340)
Supplement: Supplementary file 1 — Supplementary [file CMDC-12-1542-s001.pdf]

CHEMMEDCHEM
Chemistry & Drug Discovery

Supporting Information

Identification of a Potent Phosphoinositide 3-Kinase Pan Inhibitor Displaying a Strategic Carboxylic Acid Group and Development of Its Prodrugs

Tracey Pirali+,[a] Elisa Ciraolo+,[b] Silvio Aprile,[a] Alberto Massarotti,[a] Alex Berndt,[c] Alessia Griglio,[a] Marta Serafini,[a] Valentina Mercalli,[a] Clarissa Landoni,[a] Carlo Cosimo Campa,[b] Jean Piero Margaria,[b] Rangel L. Silva,[d] Giorgio Grosa,[a] Giovanni Sorba,[a] Roger Williams,[c] Emilio Hirsch,*[b, e] and Gian Cesare Tron*[a, e]

cmdc_201700340_sm_miscellaneous_information.pdf

ToC:

p. S2 Chemistry

p. S14 Biology

p. S15 Crystallography

p. S17 Metabolism

p. S24 References

Chemistry

5-(bis(Methylthio)methylene)-2,2-dimethyl-1,3-dioxane-4,6-dione (16). ^{1}H NMR (300 MHz, $CDCl_3$): δ = 2.64 (s, 6H), 1.73 (s, 6H) ppm; MS (ESI) *m/z* 249 $[M+H]^+$.

5-((2-Iodophenylamino)(methylthio)methylene)-2,2-dimethyl-1,3-dioxane-4,6-dione (18). ^{1}H NMR (300 MHz, $CDCl_3$): δ = 7.88 (d, *J* = 7.4 Hz, 1H), 7.48-7.32 (m, 2H), 7.04 (t, *J* = 7.4 Hz, 1H), 2.18 (s, 3H), 1.73 (s, 6H) ppm; ^{13}C NMR (300 MHz, $CDCl_3$): δ = 180.1, 141.8, 141.3, 131.1, 130.9, 128.9, 104.8, 98.2, 88.9, 59.8, 27.9, 20.3 ppm; MS (ESI) *m/z* 420 $[M+H]^+$.

5-((2-Iodophenylamino)(morpholino)methylene)-2,2-dimethyl-1,3-dioxane-4,6-dione (20). ^{1}H NMR (300 MHz, CDC_{l3}): δ = 9.57 (br s, 1H), 7.90 (d, *J* = 7.7 Hz, 1H), 7.40 (t, *J* = 7.7 Hz, 1H), 7.10 (d, *J* = 7.7 Hz, 1H), 7.01 (t, *J* = 7.7 Hz, 1H), 3.62 (t, *J* = 4.4 Hz, 4H), 3.18 (t, *J* = 4.4 Hz, 4H), 1.74 (s, 6H) ppm; ^{13}C NMR (300 MHz, $CDCl_3$): δ = 166.2, 165.9, 142.7, 141.9, 130.9, 130.1, 127.6, 103.9, 130.1, 127.6, 103.9, 97.6, 78.5, 66.7, 52.3, 28.0 ppm. MS (ESI) *m/z* 457 $[M-H]^-$.

8-Iodo-2-morpholinoquinolin-4(1H)-one (21). ^{1}H NMR (300 MHz, $CDCl_3$): δ = 8.18 (d, *J* = 6.6 Hz, 1H), 8.00 (d, *J* = 6.6 Hz, 1H), 6.98 (t, *J* = 6.6 Hz, 1H), 5.77 (br s, 1H), 3.95-3.72 (m, 5H), 3.53-3.20 (m, 4H) ppm; ^{13}C NMR (300 MHz, $CDCl_3$): δ = 163.9, 160.7, 149.0, 141.6, 124.5, 124.4, 119.1, 102.5, 92.9, 68.0, 46.9 ppm; MS (ESI) *m/z* 357 $[M+H]^+$.

2-Morpholino-8-((trimethylsilyl)ethynyl) quinolin-4(1H)-one (22). ^{1}H NMR (300 MHz, $CDCl_3$): δ = 8.12 (d, *J* = 7.4, 1H), 7.59 (d, *J* = 7.4 Hz, 1H), 7.11 (t, *J* = 7.4 Hz, 1H), 5.86 (br s, 1H), 3.77 (t, *J* = 4.7 Hz, 4H), 3.34 (t, *J* = 4.7 Hz, 4H), 0.24 (s, 9H) ppm; MS (ESI) *m/z* 327 $[M+H]^+$.

8-Ethynyl-2-morpholinoquinolin-4(1H)-one (12). ^{1}H NMR (300 MHz, $CDCl_3$): δ = 8.19 (d, *J* = 7.4 Hz, 1H), 7.66 (d, *J* = 7.4 Hz, 1H), 7.17 (t, *J* = 7.4 Hz, 1H), 5.90 (br s, 1H), 3.79 (t, *J* = 4.9 Hz, 4H), 3.61 (s, 1H), 3.37 (t, *J* = 4.9 Hz, 4H) ppm; MS (ESI) *m/z* 255 $[M+H]^+$.

Synthesis of 4-morpholino-4H-pyrrolo[3,2,1-ij]quinolin-6(5H)-one (24). ^{1}H NMR (300 MHz, $[D_6]$DMSO): δ = 7.62 (d, *J* = 7.4 Hz, 1H), 7.50 (d, *J* = 7.4 Hz, 1H), 7.20 (d, *J* = 3.6 Hz, 1H), 7.07 (t, *J* = 7.4 Hz, 1H), 6.50 (d, *J* = 3.6 Hz, 1H), 5.47 (s, 1H), 3.94 (t, *J* = 4.5 Hz, 4H), 3.29 (t, *J* = 4.5 Hz, 4H) ppm; MS (ESI) *m/z* 257 $[M+H]^+$.

Characterization of compounds 26-53.

8-(1-(3-Hydroxyphenyl)-1H-1,2,3-triazol-4-yl)-2-morpholinoquinolin-4(1H)-one (26). ^{1}H NMR (300 MHz, [D$_6$]DMSO): δ = 9.25 (s, 1H), 8.37 (br s, 1H), 7.95 (d, J = 7.7 Hz, 1H), 7.39 (t, J = 7.7 Hz, 1H), 7.29-7.26 (m, 4H), 6.86 (d, J = 7.9 Hz, 1H), 6.49 (s, 1H), 3.75-3.72 (m, 4H), 3.54-3.50 (m, 4H) ppm; MS (ESI) m/z 389 [M+H]$^+$; Anal. calcd for $C_{21}H_{19}N_5O_3$: C, 64.77; H, 4.92; N, 17.98, found: C, 64.54; H, 4.72; N, 18.05.

8-(1-(3-Hydroxy-4-methoxyphenyl)-1H-1,2,3-triazol-4-yl)-2-morpholinoquinolin-4(1H)-one (27). ^{1}H NMR (300 MHz, [D$_6$]DMSO): δ = 9.18 (s, 1H), 8.47 (br s, 1H), 8.00 (s, 1H), 7.33-7.30(m, 4H), 7.17 (d, J = 8.8 Hz, 1H), 6.51 (s, 1H), 3.86 (s, 3H), 3.75-3.70 (m, 4H), 3.57-3.54 (m, 4H) ppm; MS (ESI) m/z 420 [M+H]$^+$; Anal. calcd for $C_{22}H_{21}N_5O_4$: C, 63.00; H, 5.05; N, 16.70, found: C, 63.01; H, 5.32; N, 16.68.

8-(1-(4-Chlorophenyl)-1H-1,2,3-triazol-4-yl)-2-morpholinoquinolin-4(1H)-one (28). ^{1}H NMR (300 MHz, [D$_6$]DMSO): δ = 8.48 (s, 1H), 7.98-7.86 (m, 4H), 7.73 (d, J = 8.3 Hz, 2H), 7.34 (t, J = 7.7 Hz, 1H), 6.50 (s, 1H), 3.80-3.77 (m, 4H), 3.59-3.56 (m, 4H) ppm; MS (ESI) m/z 408 [M+H]$^+$; Anal. calcd for $C_{21}H_{18}ClN_5O_2$: C, 61.84; H, 4.45; N, 17.17, found: C, 62.05; H, 4.64; N, 17.32.

2-Morpholino-8-(1-(4-(trifluoromethoxy) phenyl)-1H-1,2,3-triazol-4-yl)quinolin-4(1H)-one (29). ^{1}H NMR (300 MHz, [D$_6$]DMSO): δ = 9.38 (s, 1H), 8.45 (d, J 7.7 Hz, 1H), 8.10-8.03 (m, 4H), 7.69 (d, J = 7.7 Hz, 1H), 7.35 (t, J = 7.7 Hz, 1H), 6.55 (s, 1H), 3.83-3.79 (m, 4H), 3.64-3.57 (m, 4H) ppm; MS (ESI) m/z 458 [M+H]$^+$; Anal. calcd for $C_{22}H_{18}F_3N_5O_3$: C, 57.77; H, 3.97; N, 15.31, found: C, 58.09; H, 4.21; N, 15.21.

8-(1-(4-Methoxy-3-nitrophenyl)-1H-1,2,3-triazol-4-yl)-2-morpholinoquinolin-4(1H)-one (30). ^{1}H NMR (300 MHz, [D$_6$]DMSO): δ = 9.40 (s, 1H), 8.47 (s, 1H), 8.25 (d, J = 9.0 Hz, 1H), 8.00 (d, J = 9.0 Hz, 1H), 7.65-7.62 (m, 2H), 7.34 (t, J = 9.0 Hz, 1H), 6.53 (s, 1H), 4.03 (s, 3H), 3.80-3.76 (m, 4H), 3.60-3.54 (m, 4H) ppm; MS (ESI) m/z 449 [M+H]$^+$; Anal. calcd for $C_{22}H_{20}N_6O_5$: C, 58.92; H, 4.50; N, 18.74, found: C, 58.89; H, 4.75; N, 18.52.

Methyl-4-((4-(2-morpholino-4-oxo-1,4-dihydroquinolin-8-yl)-1H-1,2,3-triazol-1-yl)methyl)benzoate (31). ^{1}H NMR (300 MHz, [D$_6$]DMSO): δ = 8.72 (s, 1H), 8.36 (br s, 1H), 7.91-7.87(m, 4H) 7.52 (d, J = 8.2 Hz, 2H), 7.26 (t, J = 7.6 Hz, 1H), 6.47 (s, 1H), 5.81 (s, 2H), 3.95 (s, 3H), 3.66-3.60 (m, 4H), 3.56-3.51 (m, 4H) ppm; MS (ESI) m/z 446 [M-H]$^-$; Anal. calcd for $C_{24}H_{23}N_5O_4$: C, 64.71; H, 5.20; N, 15.72, found: C, 64.48; H, 5.42; N, 15.71.

8-(1-(4-Aminophenyl)-1H-1,2,3-triazol-4-yl)-2-morpholinoquinolin-4(1H)-one (32). ^{1}H NMR (300 MHz, [D$_6$]DMSO): *δ* = 9.09 (s, 1H), 8.42 (br s, 2H), 7.95-7.93 (m, 4H), 7.50 (d, *J* = 8.5 Hz, 1H), 7.32 (t, *J* = 8.5 Hz, 1H), 6.75 (d, *J* = 8.5 Hz, 1H), 6.62 (s, 1H), 3.82-3.78 (m, 4H), 3.58-3.54 (m, 4H) ppm; MS (ESI) *m/z* 389 $[M+H]^+$; Anal. calcd for $C_{21}H_{20}N_6O_2$: C, 64.94; H, 5.19; N, 21.64, found: C, 65.12; H, 5.36; N, 21.86.

2-(4-(4-(2-Morpholino-4-oxo-1,4-dihydroquinolin-8-yl)-1H-1,2,3-triazol-1-yl)phenyl) acetonitrile (33). ^{1}H NMR (300 MHz, [D$_6$]DMSO): *δ* = 8.39 (s, 1H), 7.96-7.93 (m, 4H), 7.60 (d, *J* = 8.5 Hz, 2H), 7.30 (t, *J* = 8.5 Hz, 1H), 6.55 (s, 1H), 4.14 (s, 2H), 3.77-3.72 (m, 4H), 3.55-3.52 (m, 4H) ppm; MS (ESI) *m/z* 413 $[M+H]^+$; Anal. calcd for $C_{23}H_{20}N_6O_2$: C, 66.98; H, 4.89; N, 20.38, found: C, 67.23; H, 5.12; N, 20.05.

2-Morpholino-8-(1-(naphthalen-2-yl)-1H-1,2,3-triazol-4-yl)quinolin-4(1H)-one (34). ^{1}H NMR (300 MHz, [D$_6$]DMSO): *δ* = 9.56 (s, 1H), 8.54-8.52 (m, 2H), 8.27-8.11 (m, 5H), 7.71-7.69 (m, 2H), 7.38 (t, *J* = 7.9 Hz, 1H), 6.61 (s, 1H), 3.80-3.76 (m, 4H), 3.65-3.59 (m, 4H) ppm; MS (ESI) *m/z* 424 $[M+H]^+$; Anal. calcd for $C_{25}H_{21}N_5O_2$: C, 70.91; H, 5.00; N, 16.54, found: C, 71.23; H, 5.32; N, 16.76.

2-Morpholino-8-(1-(quinolin-3-yl)-1H-1,2,3-triazol-4-yl)quinolin-4(1H)-one (36). ^{1}H NMR (300 MHz, [D$_6$]DMSO): *δ* = 9.53 (s, 1H), 8.98-8.96 (m, 2H), 8.40 (s, 1H), 8.21-8.16 (m, 2H), 7.97 (s, 1H), 7.90 (t, *J* = 7.1 Hz, 1H), 7.77 (t, *J* = 7.4 Hz, 1H), 7.36 (t, *J* = 7.1 Hz, 1H), 6.52 (s, 1H), 3.82-3.80 (m, 4H), 3.66-3.62 (m, 4H) ppm; MS (ESI) *m/z* 425 $[M+H]^+$; Anal. calcd for $C_{24}H_{20}N_6O_2$: C, 67.91; H, 4.75; N, 19.80, found: C, 68.00; H, 4.82; N, 19.83.

N-(4-Methoxy-3-(4-(2-morpholino-4-oxo-1,4-dihydroquinolin-8-yl)-1H-1,2,3-triazol-1-yl)phenyl)acetamide (38). ^{1}H NMR (300 MHz, [D$_6$]DMSO): *δ* = 10.1 (br s, 1H), 9.25 (s, 1H), 8.53 (d, *J* = 6.8 Hz, 1H), 8.13 (d, *J* = 2.5 Hz, 1H), 8.00 (d, *J* = 6.8 Hz, 1H), 7.67 (dd, *J* = 8.8, 2.5 Hz, 1H), 7.34-7.31 (m, 2H), 6.55 (s, 1H), 3.88 (s, 3H), 3.81-3.79 (m, 4H), 3.59-3.57 (m, 4H), 2.06 (s, 3H) ppm; MS (ESI) *m/z* 461 $[M+H]^+$; Anal. calcd for $C_{24}H_{24}N_6O_4$: C, 62.60; H, 5.25; N, 18.25, found: C, 62.82; H, 5.47; N, 18.01.

8-(1-(3-Methoxy-5-(trifluoromethoxy)phenyl)-1H-1,2,3-triazol-4-yl)-2-morpholinoquinolin-4(1H)-one (39). ^{1}H NMR (300 MHz, [D$_6$]DMSO): *δ* = 9.52 (s, 1H), 8.43 (d, *J* = 7.7 Hz, 1H), 8.00 (d, *J* = 7.7 Hz, 1H), 7.85-7.83 (m, 2H), 7.41 (s, 1H), 7.35 (t, *J* = 7.7 Hz, 1H), 6.55 (s, 1H), 3.98 (s, 3H), 3.78-3.76 (m, 4H), 3.63-3.61 (m, 4H) ppm;

MS (ESI) *m/z* 472 [M+H]$^+$; Anal. calcd for $C_{23}H_{20}F_3N_5O_4$: C, 56.67; H, 4.14; N, 14.37, found: C, 56.42; H, 4.14; N, 14.57.

8-(1-(4-Hydroxy-2-methylphenyl)-1H-1,2,3-triazol-4-yl)-2-morpholinoquinolin-4(1H)-one (40). ^{1}H NMR (300 MHz, [D$_6$]DMSO): δ = 8.86 (s, 1H), 8.43 (br s, 1H), 7.99 (s, 1H), 7.33-7.31 (m, 2H), 6.86-6.79 (m, 3H), 6.70 (s, 1H), 3.75-3.73 (m, 4H), 3.53-3.51 (m, 4H), 2.14 (s, 3H) ppm; MS (ESI) *m/z* 404 [M+H]$^+$; Anal. calcd for $C_{22}H_{21}N_5O_3$: C, 65.50; H, 5.25; N, 17.36, found: C, 65.77; H, 5.20; N, 17.56.

8-(1-(2-(Hydroxymethyl)phenyl)-1H-1,2,3-triazol-4-yl)-2-morpholinoquinolin-4(1H)-one (41). ^{1}H NMR (300 MHz, [D$_6$]DMSO): δ = 9.16 (s, 1H), 8.47 (br s, 1H), 7.95-7.93 (m, 2H), 7.55-7.52 (m, 2H), 7.33-7.30 (m, 2H), 7.03 (t, *J* = 7.4 Hz, 1H), 6.60 (s, 1H), 4.46 (s, 2H), 3.74-3.70 (m, 4H), 3.60-3.56 (m, 4H) ppm; MS (ESI) *m/z* 404 [M+H]$^+$; Anal. calcd for $C_{22}H_{21}N_5O_3$: C, 65.50; H, 5.25; N, 17.36, found: C, 65.52; H, 5.25; N, 17.38.

Methyl 4-(4-(2-morpholino-4-oxo-1,4-dihydroquinolin-8-yl)-1H-1,2,3-triazol-1-yl)benzoate (42). ^{1}H NMR (300 MHz; [D$_6$]DMSO): δ = 9.40 (s, 1H), 8.42 (d, *J* = 7.7 Hz, 1H), 8.24-8.22 (m, 2H) 8.10-8.07 (m, 3H), 7.35 (t, *J* = 7.7 Hz, 1H), 6.48 (s, 1H), 3.94 (s, 3H), 3.84-3.82 (m, 4H), 3.65-3.61 (m, 4H) ppm; MS (ESI) *m/z* 432 [M+H]$^+$; Anal. calcd for $C_{23}H_{21}N_5O_4$: C, 64.03; H, 4.91; N, 16.23, found: C, 64.32; H, 5.13; N, 16.47.

8-(1-(3,4-Dimethoxyphenyl)-1H-1,2,3-triazol-4-yl)-2-morpholinoquinolin-4(1H)-one (43). ^{1}H NMR (300 MHz, [D$_6$]DMSO): δ = 9.29 (s, 1H), 8.43 (d, *J* = 7.7 Hz, 1H), 7.98 (d, *J* = 7.7 Hz, 1H), 7.51 (s, 1H), 7.43 (d, *J* = 8.5 Hz, 1H), 7.33 (t, *J* = 7.7 Hz, 1H), 7.20 (d, *J* = 8.5 Hz, 1H), 6.55 (s, 1H), 3.90 (s, 3H), 3.85 (s, 3H), 3.79-7.77 (m, 4H), 3.64-3.60 (m, 4H) ppm; MS (ESI) *m/z* 434 [M+H]$^+$; Anal. calcd for $C_{23}H_{23}N_5O_4$: C, 63.73; H, 5.35; N, 16.16, found: C, 64.12; H, 5.70; N, 15.98.

8-(1-(4-Methoxyphenyl)-1H-1,2,3-triazol-4-yl)-2-morpholinoquinolin-4(1H)-one (44). ^{1}H NMR (300 MHz, [D$_6$]DMSO): δ = 9.21 (s, 1H), 8.39 (br s, 1H), 8.00 (d, *J* = 7.9 Hz, 1H), 7.85 (d, *J* = 7.7 Hz, 2H), 7.33 (t, *J* = 7.9 Hz, 1H), 7.21-7.19 (m, 3H), 6.58 (s, 1H), 3.86 (s, 3H), 3.80-3.78 (m, 4H), 3.59-3.56 (m, 4H) ppm; MS (ESI) *m/z* 404 [M+H]$^+$; Anal. calcd for $C_{22}H_{21}N_5O_3$: C, 65.50; H, 5.25; N, 17.36, found: C, 65.82; H, 5.43; N, 17.21.

8-(1-(Benzo[d][1,3]dioxol-5-yl)-1H-1,2,3-triazol-4-yl)-2-morpholinoquinolin-4(1H)-one (46). ^{1}H NMR (300 MHz, [D$_6$]DMSO): δ = 9.23 (s, 1H), 8.42 (br s, 1H), 8.01-7.98 (m, 2H), 7.54 (s, 1 H), 7.33 (t, *J* = 7.7 Hz, 1H),

7.18-7.15 (m, 2H), 6.58 (s, 1H), 6.19 (s, 2H), 3.79-3.77 (m, 4H), 3.59-3.57 (m, 4H) ppm; MS (ESI) *m/z* 418 $[M+H]^+$; Anal. calcd for $C_{22}H_{19}N_5O_4$: C, 63.30; H, 4.59; N, 16.78, found: C, 63.57; H, 4.73; N, 16.53.

8-(1-(3,5-Dimethoxyphenyl)-1H-1,2,3-triazol-4-yl)-2-morpholinoquinolin-4(1H)-one (47). ^1H NMR (300 MHz, $[D_6]DMSO$): δ = 9.46 (s, 1H), 8.48 (br s, 1H), 8.07 (s, 1H), 7.45 (t, *J* = 7.6 Hz, 1H), 7.21-7.17 (m, 2H), 6.78 (s, 2H), 6.72 (s, 1H), 3.86 (s, 6H) 3.79-3.77 (m, 4H), 3.66-3.64 (m, 4H) ppm; MS (ESI) *m/z* 434 $[M+H]^+$; Anal. calcd for $C_{23}H_{23}N_5O_4$: C, 63.73; H, 5.35; N, 16.16, found: C, 64.02; H, 5.62; N, 16.21.

Methyl 3-(4-(2-morpholino-4-oxo-1,4-dihydroquinolin-8-yl)-1H-1,2,3-triazol-1-yl)benzoate (49). ^1H NMR (300 MHz, $[D_6]DMSO$): δ = 8.41-8.39 (m, 2H), 8.27 (d, *J* = 7.1 Hz, 1H), 8.10-7.99 (m, 3H), 7.82 (t, *J* = 7.9 Hz, 1H), 7.35 (t, *J* = 7.1 Hz, 1H), 6.53 (s, 1H), 4.01 (s, 3H), 3.84-3.82 (m, 4H), 3.62-3.60 (m, 4H) ppm; MS (ESI) *m/z* 432 $[M+H]^+$; Anal. calcd for $C_{23}H_{21}N_5O_4$: C, 64.03; H, 4.91; N, 16.23, found: C, 64.11; H, 4.90; N, 16.00.

4-(4-(2-Morpholino-4-oxo-1,4-dihydroquinolin-8-yl)-1H-1,2,3-triazol-1-yl)benzenesulfonamide (50). ^1H NMR (300 MHz, $[D_6]DMSO$): δ = 8.44 (s, 1H), 8.18-8.00 (m, 4H), 7.56-7.54 (m, 2H), 7.35 (t, *J* = 7.7 Hz, 1H), 6.65 (s, 1H), 3.81-3.79 (m, 4H), 3.61-3.59 (m, 4H) ppm; MS (ESI) *m/z* 453 $[M+H]^+$; Anal. calcd for $C_{21}H_{20}N_6O_4S$: C, 55.74; H, 4.46; N, 18.57, found: C, 55.94; H, 4.69; N, 18.70.

8-(1-(2-Methoxyphenyl)-1H-1,2,3-triazol-4-yl)-2-morpholinoquinolin-4(1H)-one (51). ^1H NMR (300 MHz, $[D_6]DMSO$): δ = 9.23 (s, 1H), 8.52 (d, *J* = 7.7 Hz, 1H), 7.99 (t, *J* = 7.4 Hz, 1H), 7.77 (d, *J* = 7.7 Hz, 1H), 7.53 (t, *J* = 7.4 Hz, 1H), 7.38-7.34 (m, 2H), 7.20 (t, *J* = 7.7 Hz, 1H), 6.54 (s, 1H), 3.91 (s, 3H), 3.83-3.80 (m, 4H), 3.60-3.56 (m, 4H) ppm; MS (ESI) *m/z* 404 $[M+H]^+$; Anal. calcd for $C_{22}H_{21}N_5O_3$: C, 65.50; H, 5.25; N, 17.36, found: C, 65.23; H, 5.25; N, 17.36.

3-(4-(2-Morpholino-4-oxo-1,4-dihydroquinolin-8-yl)-1H-1,2,3-triazol-1-yl)benzoic acid (52). ^1H NMR (300 MHz, $[D_6]DMSO$): δ = 9.42 (s, 1H), 8.42-8.40 (m, 2H), 8.16 (d, *J* = 8.2 Hz, 1H), 8.09-8.04 (m, 2H), 7.76 (t, *J* = 8.2 Hz, 1H), 7.34 (t, *J* = 7.7 Hz, 1H), 6.58 (s, 1H), 3.87-3.85 (m, 4H), 3.65-3.63 (m, 4H) ppm; MS (ESI) *m/z* 418 $[M+H]^+$; Anal. calcd for $C_{22}H_{19}N_5O_4$: C, 63.30; H, 4.59; N, 16.78, found: C, 63.36; H, 4.61; N, 16.80.

2-Morpholino-8-(1-(naphthalen-1-yl)-1H-1,2,3-triazol-4-yl)quinolin-4(1H)-one (53). ^1H NMR (300 MHz, $[D_6]DMSO$): δ = 9.56 (br s, 1H), 9.21 (s, 1H), 8.50 (d, *J* = 6.0 Hz, 1H), 8.27-8.10 (m, 2H), 8.01 (d, *J* = 7.9 Hz, 1H), 7.85 (d, *J* = 7.4 Hz, 1H), 7.78-7.69 (m, 4H), 7.37 (t, *J* = 7.7 Hz, 1H), 6.54 (s, 1H), 3.80-3.76 (m, 4H), 3.65-

3.59 (m, 4H) ppm; MS (ESI) *m/z* 424 $[M+H]^+$; Anal. calcd for $C_{25}H_{21}N_5O_2$: C, 70.91; H, 5.00; N, 16.54, found: C, 71.13; H, 5.23; N, 16.32.

Characterization of compounds 55-62.

4-(4-(2-Morpholino-4-oxo-1,4-dihydroquinolin-8-yl)-1H-1,2,3-triazol-1-yl)butanoic acid (55).

^{1}H NMR (300 MHz, [D$_6$]DMSO): δ = 8.81 (s, 1H), 8.39 (d, *J* = 7.7 Hz, 1H), 7.98 (d, *J* = 7.7 Hz, 1H), 7.35 (t, *J* = 7.7 Hz, 1H), 6.60 (s, 1H), 4.56 (t, *J* = 7.1 Hz, 2H), 3.83-3.74 (m, 4H), 3.59-3.52 (m, 4H), 2.34 (t, *J* = 7.1 Hz, 2H), 2.15 (quint, *J* = 7.1 Hz, 2H) ppm; MS (ESI) *m/z* 384 $[M+H]^+$; Anal. calcd for $C_{19}H_{21}N_5O_4$: C, 59.52; H, 5.52; N, 18.27, found: C, 59.62; H, 5.55; N, 18.17.

5-(4-(2-Morpholino-4-oxo-1,4-dihydroquinolin-8-yl)-1H-1,2,3-triazol-1-yl)pentanoic acid (56).

^{1}H NMR (300 MHz, [D$_6$]DMSO): δ = 8.79 (s, 1H), 8.29 (br s, 1H), 7.95 (d, *J* = 7.7 Hz, 1H), 7.73 (d, *J* = 7.7 Hz, 1H), 7.29 (t, *J* = 7.7 Hz, 1H), 6.53 (s, 1H), 4.50 (t, *J* = 7.4 Hz, 2H), 3.78-3.75 (m, 4H), 3.61-3.56 (m, 4H), 2.27 (t, *J* = 7.4 Hz, 2H), 1.89 (quint, *J* = 7.4 Hz, 2H), 1.52 (quint, *J* = 7.4 Hz, 2H) ppm; MS (ESI) *m/z* 398 $[M+H]^+$; Anal. calcd for $C_{20}H_{23}N_5O_4$: C, 60.44; H, 5.83; N, 17.62, found: C, 60.42; H, 5.85; N, 17.67.

6-(4-(2-Morpholino-4-oxo-1,4-dihydroquinolin-8-yl)-1H-1,2,3-triazol-1-yl)hexanoic acid (57).

^{1}H NMR (300 MHz, [D$_6$]DMSO): δ = 8.77 (s, 1H), 8.29 (d, *J* = 7.1 Hz, 1H), 7.95 (d, *J* = 7.1 Hz, 1H), 7.32 (t, *J* = 7.1 Hz, 1H), , 6.58 (s, 1H), 4.49 (t, *J* = 6.9 Hz, 2H), 3.81-3.77 (m, 4H), 3.56-3.51 (m, 4H), 2.21 (t, *J* = 6.9 Hz, 2H), 1.90-1.88 (m, 2H), 1.56-1.54 (m, 2H), 1.33-1.29 (m, 2H) ppm; MS (ESI) *m/z* 412 $[M+H]^+$; Anal. calcd for $C_{21}H_{25}N_5O_4$: C, 61.30; H, 6.12; N, 17.02, found: C, 61.11; H, 5.89; N, 17.23.

***N*-(Methylsulfonyl)-3-((4-(2-morpholino-4-oxo-1,4-dihydroquinolin-8-yl)-1H-1,2,3-triazol-1-yl)methyl)benzamide (58).**

^{1}H NMR (300 MHz, [D$_6$]DMSO): δ = 8.71 (s, 1H), 8.34 (d, *J* = 8.0 Hz, 1H), 8.00 (s, 1H), 7.95-7.92 (m, 2H), 7.64 (d, *J* = 8.0 Hz, 1H), 7.58 (t, *J* = 8.0 Hz, 1H), 7.27 (t, *J* = 7.3 Hz, 1H), 6.48 (s, 1H), 5.80 (s, 2H), 3.67-3.63 (m, 4H), 3.35-3.31 (m, 4H), 2.98 (s, 3H) ppm; MS (ESI) *m/z* 509 $[M+H]^+$; Anal. calcd for $C_{24}H_{24}N_6O_5S$: C, 56.68; H, 4.76; N, 16.53, found: C, 56.89; H, 4.99; N, 16.54.

3-((4-(2-Morpholino-4-oxo-1,4-dihydroquinolin-8-yl)-1H-1,2,3-triazol-1-yl)methyl)benzamide (59).

^{1}H NMR (300 MHz, [D$_6$]DMSO): δ = 8.69 (s, 1H), 8.36 (d, *J* = 6.9 Hz, 1H), 8.07 (s, 1H), 7.95 (br s, 2H), 7.92-7.87 (m, 2H), 7.59-7.47 (m, 2H), 7.27 (t, *J* = 8.0 Hz, 1H), 6.48 (s, 1H), 5.80 (s, 2H), 3.67-3.63 (m, 4H), 3.35-

3.31 (m, 4H) ppm; MS (ESI) *m/z* 431 $[M+H]^+$; Anal. calcd for $C_{23}H_{22}N_6O_3$ requires: C, 64.17; H, 5.15; N, 19.52, found: C, 64.36; H, 5.11; N, 19.42.

***N*-Benzyl-3-((4-(2-morpholino-4-oxo-1,4-dihydroquinolin-8-yl)-1H-1,2,3-triazol-1-yl)methyl)benzamide (60).**

^{1}H NMR (300 MHz, $[D_6]$DMSO): δ = 9.13 (br s, 1H), 8.70 (s, 1H), 8.36 (br s, 1H), 7.98-7.91 (m, 3H), 7.58-7.48 (m, 2H), 7.31-7.23 (m, 7H), 6.52 (s, 1H), 5.78 (s, 2H), 4.47 (d, *J* = 7.7 Hz, 2H), 3.67-3.61 (m, 4H), 3.35-3.30 (m, 4H) ppm; MS (ESI) *m/z* 521 $[M+H]^+$; Anal. calcd for $C_{30}H_{28}N_6O_3$: C, 69.22; H, 5.42; N, 16.14, found: C, 69.31; H, 5.46; N, 16.16.

***N*-Cyclopropyl-3-((4-(2-morpholino-4-oxo-1,4-dihydroquinolin-8-yl)-1H-1,2,3-triazol-1-yl)methyl)benzamide (61).**

^{1}H NMR (300 MHz, $[D_6]$DMSO): δ = 8.53 (s, 1H), 8.36 (d, *J* = 6.9 Hz, 1H), 8.03-7.86 (m, 3H), 7.59-7.47 (m, 2H), 7.30 (t, *J* = 8.0 Hz, 1H), 6.52 (s, 1H), 5.75 (s, 2H), 3.67-3.63 (m, 4H), 3.35-3.32 (m, 4H), 2.80-2.77 (m, 1H), 0.69-0.66 (m, 2H), 0.58-0.56 (m, 2H) ppm; MS (ESI) *m/z* 471 $[M+H]^+$; Anal. calcd for $C_{26}H_{26}N_6O_3$: C, 66.37; H, 5.57; N, 17.86, found: C, 66.22; H, 5.60; N, 17.62.

***N,N*-Diethyl-3-((4-(2-morpholino-4-oxo-1,4-dihydroquinolin-8-yl)-1H-1,2,3-triazol-1-yl)methyl)benzamide (62).**

^{1}H NMR (300 MHz, $[D_6]$DMSO): δ = 9.06-9.03 (m, 2H), 8.75 (s, 1H), 8.00 (d, *J* = 7.1 Hz, 1H), 7.88-7.84 (m, 2H), 7.74 (d, *J* = 7.1 Hz, 1H), 7.61 (t, *J* = 7.1 Hz, 1H), 6.50 (s, 1H), 5.77 (s, 2H), 3.88-3.86 (m, 2H), 3.70-3.66 (m, 4H), 3.35-3.31 (m, 4H), 3.13-3.10 (m, 2H), 1.89-1.85 (m, 3H), 1.71-1,67 (m, 3H) ppm; MS (ESI) *m/z* 487 $[M+H]^+$; Anal. calcd for $C_{27}H_{30}N_6O_3$: C, 66.65; H, 6.21; N, 17.27, found: C, 66.86; H, 6.57; N, 16.96.

8-(1-(3-(1H-tetrazol-5-yl)benzyl)-1H-1,2,3-triazol-4-yl)-2-morpholinoquinolin-4(1H)-one (63).

^{1}H NMR (300 MHz, $[D_6]$DMSO): δ = 8.77 (s, 1H), 8.30 (d, *J* = 7.1, 1H), 7.95 (s, 1H), 7.44-7.40 (m, 2H), 7.30 (d, *J* = 7.1 Hz, 1H), 7.24 (t, *J* = 7.1 Hz, 1H), 7.16 (t, *J* = 7.5 Hz, 1H), 6.87 (s, 1H), 5.76 (s, 2H), 3.63-3.59 (m, 4H), 3.38-3.32 (m, 4H) ppm; MS (ESI) *m/z* 456 $[M+H]^+$; Anal. calcd for $C_{23}H_{21}N_9O_2$: C, 60.65; H, 4.65; N, 27.68, found: 60.32; H, 4.29; N, 27.78.

Characterization of prodrugs 65-76.

Benzyl 3-((4-(2-morpholino-4-oxo-1,4-dihydroquinolin-8-yl)-1H-1,2,3-triazol-1-yl)methyl)benzoate (65). mp: 209-210°C; ^{1}H NMR (300 MHz, [D$_6$]DMSO): δ = 8.73 (s, 1H), 8.36 (d, J = 7.7 Hz, 1H), 8.04-7.93 (m, 3H), 7.71 (d, J = 7.7 Hz, 1H), 7.59 (t, J = 7.7 Hz, 1H), 7.60-7.46 (m, 2H), 7.36-7.26 (m, 4H), 6.48 (s, 1H), 5.84 (s, 2H), 5.35 (s, 2H), 3.82-3.63 (m, 4H), 3.58-3.12 (m, 4H) ppm; IR (KBr) 3424, 2855, 1716, 1580, 1228, 787, 536, 441 cm^{-1}; MS (ESI) m/z 522 [M+H]$^+$; Anal. calcd for $C_{30}H_{27}N_5O_4$: C, 69.08; H, 5.22; N, 13.43, found: C, 69.10; H, 5.32; N, 13.38.

Isopropyl 3-((4-(2-morpholino-4-oxo-1,4-dihydroquinolin-8-yl)-1H-1,2,3-triazol-1-yl)methyl)benzoate (66). mp: 170-171°C; ^{1}H NMR (300 MHz, [D$_6$]DMSO): δ = 8.70 (s, 1H), 8.35 (d, J = 6.9 Hz, 1H), 8.01 (s, 1H), 7.96-7.92 (m, 2H), 7.68 (d, J = 6.9 Hz, 1H), 7.57 (t, J = 6.9 Hz, 1H), 7.28 (t, J = 7.6 Hz, 1H), 6.46 (s, 1H), 5.81 (s, 2H), 5.13 (hept, J = 6.3 Hz, 1H), 3.65-3.61 (m, 4H), 3.40-3.35 (m, 4H), 1.31 (d, J = 6.3 Hz, 6H) ppm; IR (KBr) 3271, 2974, 2360, 1717, 1284, 1122, 931, 792, 402 cm^{-1}; MS (ESI) m/z 474 [M+H]$^+$; Anal. calcd for $C_{26}H_{27}N_5O_4$: C, 65.95; H, 5.75; N, 14.79, found: C, 66.21; H, 6.02; N, 14.84.

Ethyl 3-((4-(2-morpholino-4-oxo-1,4-dihydroquinolin-8-yl)-1H-1,2,3-triazol-1-yl)methyl)benzoate (67). mp: 204-205°C; ^{1}H NMR (300 MHz, [D$_6$]DMSO): δ = 8.72 (s, 1H), 8.35 (br s, 1H), 8.03 (s, 1H), 7.96-7.92 (m, 3H), 7.69 (d, J = 7.7 Hz, 1H), 7.58 (t, J = 7.7 Hz, 1H), 7.28 (t, J = 7.9 Hz, 1H), 6.45 (s, 1H), 5.82 (s, 2H), 4.33 (q, J = 7.1 Hz, 2H), 3.67-3.54 (m, 4H), 3.45-3.38 (m, 4H), 1.30 (t, J = 7.1 Hz, 3H) ppm; IR (KBr) 3368, 2967, 2362, 1710, 1625, 1293, 1122, 1018, 736, 406 cm^{-1}; MS (ESI) m/z 460 [M+H]$^+$; Anal. calcd for $C_{25}H_{25}N_5O_4$: C, 65.35; H, 5.48; N, 15.24, found: C, 65.56; H, 5.77; N, 15.31.

Butyl 3-((4-(2-morpholino-4-oxo-1,4-dihydroquinolin-8-yl)-1H-1,2,3-triazol-1-yl)methyl)benzoate (68). mp: 183-185°C; ^{1}H NMR (300 MHz, [D$_6$]DMSO): δ = 9.08 (s, 1H), 8.72 (d, J = 7.1 Hz, 1H), 8.36 (d, J = 7.5 Hz, 1H), 7.98 (s, 1H), 7.93 (t, J = 7.1 Hz, 1H), 7.68 (d, J = 7.5, 1H), 7.58 (t, J = 7.5 Hz, 1H), 7.28 (t, J = 7.1 Hz, 1H), 6.48 (s, 1H), 5.83 (s, 2H), 4.25 (t, J = 7.4 Hz, 2H), 3.69-3.64 (m, 4H), 3.43-3.39 (m, 4H), 1.65 (quint, J = 7.4 Hz, 2H), 1.38 (sext, J = 7.4 Hz, 2H), 0.86 (t, J = 7.4 Hz, 3H) ppm; IR (KBr) 2968, 1727, 1625, 1576, 1366, 795, 728 cm^{-1}; MS (ESI) m/z 488 [M+H]$^+$; Anal. calcd for $C_{27}H_{29}N_5O_4$: C, 66.51; H, 6.00; N, 14.36, found: C, 66.43; H, 5.87; N, 14.43.

tert-Butyl 3-((4-(2-morpholino-4-oxo-1,4-dihydroquinolin-8-yl)-1H-1,2,3-triazol-1-yl)methyl)benzoate (69).

mp: 189-190°C; ^{1}H NMR (300 MHz, $CDCl_3$): δ = 8.30 (d, J =7.1 1H), 8.01 (s, 1H), 7.95 (s, 1H), 7.67 (d, J = 7.1 Hz, 1H), 7.53-7.47 (m, 2H), 7.27-7.26 (m, 2H), 6.00 (s, 1H), 5.68 (s, 2H), 3.92-3.88 (m, 4H), 3.5-3.50 (m, 4H), 1.59 (s, 9H) ppm; IR (KBr) 2974, 1716, 1626, 1587, 795, 736 cm^{-1}; MS (ESI) m/z 488 $[M+H]^+$; Anal. calcd for $C_{27}H_{29}N_5O_4$: C, 66.51; H, 6.00; N, 14.36, found: C, 66.54; H, 6.02; N, 14.35.

Pyridin-4-ylmethyl 3-((4-(2-morpholino-4-oxo-1,4-dihydroquinolin-8-yl)-1H-1,2,3-triazol-1-yl)methyl)benzoate (70).

mp: 160-161°C dec; ^{1}H NMR (300 MHz, CD_3OD): δ = 8.51 (s, 1H), 8.08 (s, 1H), 8.03 (d, J = 7.7 Hz, 1H), 7.93 (d, J = 7.7 Hz, 1H), 7.68-7.64 (m, 2H), 7.55-7.51 (m, 2H), 7.30-7.26 (m, 4H), 7.10 (t, J = 7.7 Hz, 1H), 5.71 (s, 2H), 5.32 (s, 2H), 3.81-3.77 (m, 4H), 3.43-3.36 (m, 4H) ppm; IR (KBr) 3070, 2859, 1722, 1624, 1280, 799, 737 cm^{-1}; MS (ESI) *m/z* 523 $[M+H]^+$; Anal. calcd for $C_{29}H_{26}N_6O_4$: C, 66.66; H, 5.02; N, 16.08, found: C, 66.83; H, 5.28; N, 15.99.

2-Morpholinoethyl 3-((4-(2-morpholino-4-oxo-1,4-dihydroquinolin-8-yl)-1H-1,2,3-triazol-1-yl)methyl)benzoate (71).

mp: 123-124°C; ^{1}H NMR (300 MHz, [D$_6$]DMSO): δ = 8.74 (s, 1H), 8.36 (d, J = 7.3 Hz, 1H), 7.97-7.94 (m, 3H), 7.71 (d, J = 7.5 Hz, 1H), 7.59 (t, J = 7.5 Hz, 1H), 7.28 (t, J = 7.3 Hz, 1H), 6.48 (s, 1H), 5.84 (s, 2H), 4.37 (t, J = 5.2 Hz, 2H), 3.72-3.64 (m, 8H), 3.49 (t, J = 5.2 Hz, 2H), 3.20-3.14 (m, 4H), 2.43-2.39 (m, 4H) ppm; IR (KBr) 2859, 1717, 1579, 795, 738 cm^{-1}; MS (ESI) *m/z* 545 $[M+H]^+$; Anal. calcd for $C_{29}H_{32}N_6O_5$ C, 63.96; H, 5.92; N, 15.43, found: C, 64.13; H, 6.12; N, 15.46.

Benzo[d] [1,3]dioxol-5-yl methyl 3-((4-(2-morpholino-4-oxo-1,4-dihydroquinolin-8-yl)-1H-1,2,3-triazol-1-yl)methyl)benzoate (72).

mp: 224-225°C; ^{1}H NMR (300 MHz, [D$_6$]DMSO): δ = 8.70 (s, 1H), 8.11 (d, J = 7.1 Hz, 1H), 8.03-8.00 (m, 2H), 7.93 (d, J = 6.8 Hz, 1H), 7.67 (d, J = 6.8 Hz, 1H), 7.53 (t, J = 6.8 Hz, 1H), 7.32 (t, J = 7.1 Hz, 1H), 6.90-6.88 (m, 2H), 6.74 (d, J = 8.5 Hz, 1H), 5.90 (s, 2H), 5.78 (s, 2H), 5.22 (s, 2H), 4.57 (s, 1H), 3.89-3.86 (m, 4H), 3.57-3.55

(m, 4H) ppm; IR (KBr) 3439, 2253, 1716, 1619, 1447, 764 cm^{-1}; MS (ESI) *m/z* 566 $[M+H]^+$; Anal. calcd for $C_{24}H_{23}N_5O_4$: C, 64.71; H, 5.20; N, 15.72, found: C, 64.82; H, 5.28; N, 15.65.

Isopentyl 3-((4-(2-morpholino-4-oxo-1,4-dihydroquinolin-8-yl)-1H-1,2,3-triazol-1-yl)methyl)benzoate (73).

mp: 169-170°C; ^{1}H NMR (300 MHz, [D_6]DMSO): δ = 8.73 (s, 1H), 8.37 (d, *J* = 6.8 Hz, 1H), 7.97-7.93 (m, 3H), 7.69 (d, *J* = 7.2 Hz, 1H), 7.58 (t, *J* = 7.2 Hz, 1H), 7.28 (t, *J* = 6.8 Hz, 1H), 6.49 (s, 1H), 5.83 (s, 2H), 4.27 (t, *J* = 6.3 Hz, 2H), 3.68-3.64 (m, 4H), 3.45-3.37 (m, 4H), 1.68-1.66 (m, 1H), 1.57 (quart, *J* = 6.3 Hz, 2H), 0.84 (d, *J* = 6.3 Hz, 6H) ppm; IR (KBr) 2957, 1721, 1627, 1578, 795, 730 cm^{-1}; MS (ESI) *m/z* 502 $[M+H]^+$; Anal. calcd for $C_{28}H_{31}N_5O_4$: C, 67.05; H, 6.23; N, 13.96, found: C, 67.12; H, 6.29; N, 14.00.

Pentan-2-yl 3-((4-(2-morpholino-4-oxo-1,4-dihydroquinolin-8-yl)-1H-1,2,3-triazol-1-yl)methyl)benzoate (74).

mp: 148-149°C; ^{1}H NMR (300 MHz, [D_6]DMSO): δ = 8.73 (s, 1H), 8.37 (d, *J* = 7.1 Hz, 1H), 7.97-7.93 (m, 3H), 7.68 (d, *J* = 7.6 Hz, 1H), 7.57 (t, *J* = 7.6 Hz, 1H), 7.28 (t, *J* = 7.1 Hz, 1H), 6.49 (s, 1H), 5.83 (s, 2H), 5.04 (sex, *J* = 6.3 Hz, 1H), 3.69-3.64 (m, 4H), 3.45-3.38 (m, 4H), 1.57-1.55 (m, 2H), 1.26 (d, *J* = 6.3 Hz, 3H), 1.09-1.05 (m, 2H), 0.86-0.82 (m, 3H) ppm; IR (KBr) 2962, 1715, 1624, 1578, 1459, 809, 736 cm^{-1}; MS (ESI) *m/z* 502 $[M+H]^+$; Anal. calcd for $C_{28}H_{31}N_5O_4$: C, 67.05; H, 6.23; N, 13.96, found: C, 67.28; H, 6.29; N, 14.21.

Undecyl 3-((4-(2-morpholino-4-oxo-1,4-dihydroquinolin-8-yl)-1H-1,2,3-triazol-1-yl)methyl)benzoate (75).

mp: 175-176°C; ^{1}H NMR (300 MHz, $CDCl_3$): δ = 8.36 (d, *J* = 7.1 1H), 8.07-8.03 (m, 2H), 7.97 (s, 1H), 7.63 (d, *J* = 7.1 Hz, 1H), 7.54-7.50 (m, 2H), 7.33 (br s, 1H), 7.24 (t, *J* = 7.1 Hz, 1H), 5.82 (s, 1H), 5.68 (s, 2H), 4.31 (t, *J* = 6.6 Hz, 2H), 3.93-3.88 (m, 4H) 3.51-3.45 (m, 4H), 1.74 (t, *J* = 6.6 Hz, 2H), 1.39-1.96 (m, 16H), 0.84 (t, *J* = 6.6 Hz, 3H) ppm; IR (KBr) 2923, 1722, 1577, 795, 731 cm^{-1}; MS (ESI) *m/z* 587 $[M+H]^+$; Anal. calcd for $C_{34}H_{43}N_5O_4$: C, 69.72; H, 7.40; N, 11.96, found: C, 70.12; H, 7.67; N, 12.21.

Octadecyl 3-((4-(2-morpholino-4-oxo-1,4-dihydroquinolin-8-yl)-1H-1,2,3-triazol-1-yl)methyl)benzoate (76).

mp: 186-187°C; ^{1}H NMR (300 MHz, $CDCl_3$): δ = 8.31 (d, *J* = 7.1 1H), 8.08-8.04 (m, 2H), 7.95 (s, 1H), 7.63 (d, *J* = 6.8 Hz, 1H), 7.54-7.50 (m, 2H), 7.27 (t, *J* = 7.1 Hz, 1H), 5.82 (s, 1H), 5.68 (s, 2H), 4.32 (t, *J* = 6.6 Hz, 2H), 3.95-3.87 (m, 4H) 3.55-3.46 (m, 4H), 1.82 (quint, *J* = 6.6 Hz, 2H), 1.39-1.21 (m, 30H), 0.86 (t, *J* = 6.6 Hz, 3H); IR

(KBr) 2914, 2850, 1719, 1583, 1471, 795, 731 cm^{-1}; MS (ESI) *m/z* 684 $[M+H]^+$; Anal. calcd for $C_{41}H_{57}N_5O_4$: C, 72.00; H, 8.40; N, 10.24, found: C, 72.35; H, 8.64; N, 10.47.

Biology

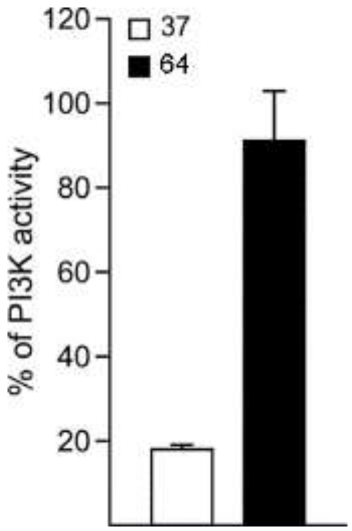

Supplementary Figure 1. 64 is not able to inhibit PI3Kα in vitro. PI3Kα recombinant protein (30 ng) was incubated with 100 nM of **37** and **64** and a mixture of PI/PS lipid micelles (1mg/mL). ATP consumption was measured and residual lipid kinase activity was indicated as percentage over untreated control.

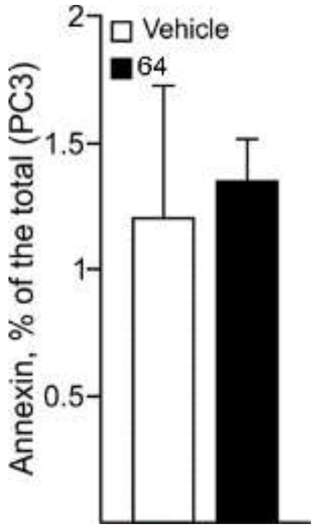

Supplementary Figure 2. **64** does not increase cell apoptosis. PC3 cells were treated with 10μM **64** and after 48h, cells were analysed by flow cytometry with FITC-annexin V. The histogram shows the percentage of positive cells over the total.

Crystallography

p110δ37 purification and crystallization

The murine p110δ construct was expressed, purified and crystals grown as previously described.[1] Briefly, a p110δ construct with a TEV protease cleavage site in a linker region between the N-terminal adaptor-binding domain (ABD) and the Ras-binding domain (RBD) of p110δ was coexpressed with the iSH2 fragment of the regulatory p85 subunit in insect cells. After an affinity purification step on Ni^{2+}-NTA resin, TEV protease was added to free the ABD/iSH2 complex from the remainder of the p110δ catalytic subunit. Following an ion-exchange chromatography step, p110δ was further purified using size-exclusion chromatography in a buffer containing 20 mM Tris pH 7.2, 50 mM $(NH_4)_2SO_4$, 1% (v/v) ethylene glycol, 1% (w/v) betaine, 0.02 % (w/v) CHAPS and 5 mM DTT. Fractions were pooled and concentrated to 4.75 mg/mL.

Initial crystals of p110δ grew in a Morpheus Screen[2] condition. p110δ37 crystals were grown by the sitting-drop vapour diffusion method at 290K in 24-well Cryschem plates by mixing 0.8 µL of the p110δ/37 mixture with 0.8 µL of a reservoir solution containing 38.6% of a GP4K solution (2X: 40% (v/v) glycerol + 20% (w/v) PEG 4000), 10 mM of a NPS solution (10X: 0.3 M of $NaNO_3$, Na_2HPO_4 and $(NH_4)_2SO_4$) and 100 mM imidazole (pH 6.8). Crystals were transferred to 2 µL of the reservoir solution and flash frozen in liquid nitrogen.

Data collection and processing

Crystals were screened and 2 datasets of p110δ37 collected from a single crystal at the ESRF beamline ID23-1 (Grenoble, France) at 100 K temperature. Diffraction images were processed using XDS.[3] For merging the datasets, SORTMTZ (CCP4 suite)[4] was used and the data scaled with SCALA.[5]

Structure determination, model building and refinement

The p110δ37 structure was solved by Molecular Replacement using Phaser from the CCP4 suite[6] and the p110δSW14 crystal structure (PDB: 2WXH) as a search model where the compound and residues near the active site were omitted to prevent model bias. The 37 structure was generated using Phenix.elbow.[7] For rebuilding and refining the model, alternating cycles of model building in Coot[8] and refinement using Refmac[9,10] were performed.

Crystallographic data

Compound Code	KIT
PDB Code	5NGB
<u>Data collection and processing</u>	
X-ray Source	ESRF ID23-1
Wavelength (Å)	0.9763
Detector	ADSC Quantum Q315r
Space group	C2221
Unit-cell parameters	
a, b, c (Å)	63.4, 143.6, 220.2
α, β, γ (º)	90.0, 90.0, 90.0
Resolution	45.63-2.90
Unique reflections	22926 (3242)
Multiplicity	10.0 (9.6)
Completeness (%)	99.8 (98.8)
Rsym	25.2 (110.4)
Rmeas	26.6 (116.9)
<I / σI>	12.1 (3.0)
<u>Refinement</u>	
Resolution (Å)	110.0-2.89
No. reflections	
work (free)	21718 (1170)
Rcryst / R free	24.2 / 29.0
Total number of atoms	
Protein	6593
Ligand	32
Deviation from ideal geometry	
Bond lengths (Å)	0.011
Bond angles (º)	1.49
Ramachandran plot:*	
Most favoured (%)	90.8
Additional allowed (%)	8.7
Generously allowed (%)	0.3
Disallowed (%)	0.1

Values in parenthesis are for the highest resolution shell
*Analysed with PROCHECK

Metabolism

Metabolic stability of PI3K inhibitors in RLM fraction

The standard incubation mixture (500 µL final volume), in 1.5 mL Eppendorf tubes, was carried out in a 50 mM TRIS-HCl buffer (pH 7.4) containing 3.3 mM $MgCl_2$, 1.3 mM $NADPNa_2$, 3.3 mM glucose 6-phosphate, 0.4 Units/mL glucose 6-phosphate dehydrogenase (NADPH regenerating system), acetonitrile as cosolvent (1% of final volume) and 50 µM of substrate. After pre-equilibration of the mixture, an appropriate volume of rat microsomal suspension was added to give a final protein concentration of 1 mg/mL. The mixture was shaken for 1 hour at 37°C and the reaction was quenched by diluting the samples with 500 µL of ice-cold acetonitrile. The samples were then centrifuged at 13,000 rpm for 10 min, and the supernatant was directly injected onto the HPLC column for analysis. Control incubations were performed without microsomes.

Stability of PI3K inhibitors in mouse plasma

An aliquot of 460 µL of blank plasma spiked with substrate (50 µM, final concentration, DMSO 5%) was incubated at 37 °C for 30 minutes. Aliquots (70 µL) of the incubation took at 0-2-5-10-20, and 30 minutes were diluted with 140 µL of acetonitrile, homogenized, and centrifuged at 13,000 rpm for five minutes. Supernatants (20 µL) were injected onto HPLC.

LC-DAD-UV

A Shimadzu HPLC system (Shimadzu, Kyoto, Japan), consisting of two LC-10AD Vp module pumps, an SLC-10A Vp system controller, an SIL-10AD Vp autosampler, and a DGU-14-A on-line degasser was used for the analysis. All the chromatographic separations were performed on a Phenomenex Luna C18 5µm (150 x 4.6 mm i.d.) (Phenomenex srl, Castel Bolognese, Italy) as a stationary phase protected by a Luna C18 SecurityGuard (Phenomenex srl). The SPD-M10Avp photodiode array detector was used to detect the analyte at 254 nm. LC Solution 1.24 software was used to process the chromatograms. Aliquots (20 µL) of supernatants obtained from incubations were injected onto the HPLC system and eluted with a mobile phase (flow rate: 1 mL/min) consisting of solvent A- 0.5% aqueous formic acid solution and solvent B- acetonitrile/methanol 80:20. The eluants were filtered through a 0.45-µm pore size polyvinylidene difluoride membrane filter before use.

The following gradients programs were used:

Compounds **64** (*Rt*= 8.8 min), **70** (*Rt*= 2.3 min), **65** (*Rt*= 13.3 min), **67** (*Rt*= 10.5 min), **66** (*Rt*= 11.8 min), **72** (*Rt*= 13.0 min), **68** (*Rt*=13.2 min), **69** (*Rt*= 12.9 min), **73** (*Rt*= 14.2 min), and **74** (*Rt*= 14.2 min): 0 min B=30%, 15 min B= 70%, 15.5 min B=30%, 20 min B=30%.
Compound **71** (*Rt*= 6.8 min): 0 min B= 20%, 15 min B= 70%, 15.5 min B= 20%, 20 min B= 20%.
Compound **75** (*Rt*=13.9 min): 0 min B= 30%, 15 min B= 95%, 15.5 min B= 30%, 20 min B=30%.
Compound **76** (*Rt*= 23.8 min): 0 min B= 30%, 7.50 min B= 50, 8.00 min B= 95%, 14.5 min B= 95%, 15.0 min B= 30%, 20 min B= 30%.

Supplementary Figure 3. Stability of **64** in rat liver microsomes (RLM), (substrate concentration: 50 µM, incubation time 60 min)

	peak area **68**	peak area **37**		% residual **68**
a) NADPH (without RLM)	484743	0		100
b) RLM (without NADPH)	156923	301263		32
c) RLM + NADPH	94724	316459		20

a)

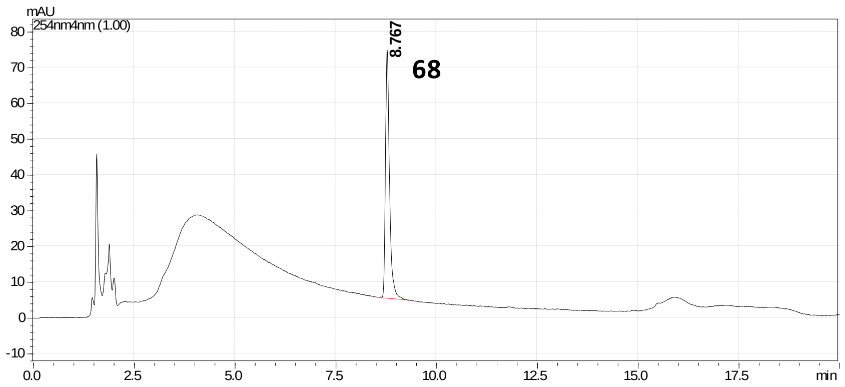

b)

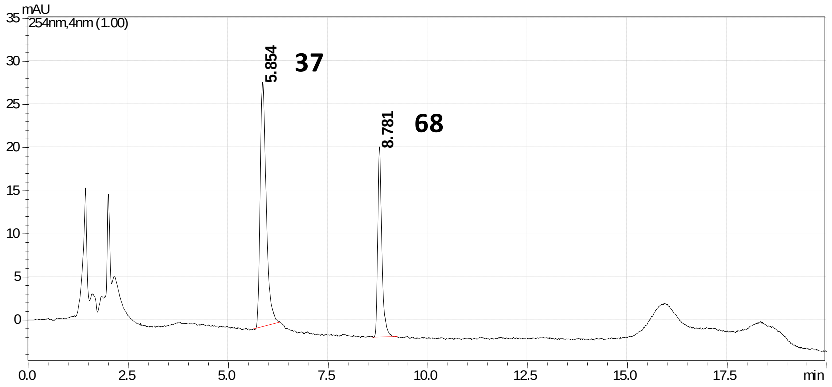

c)

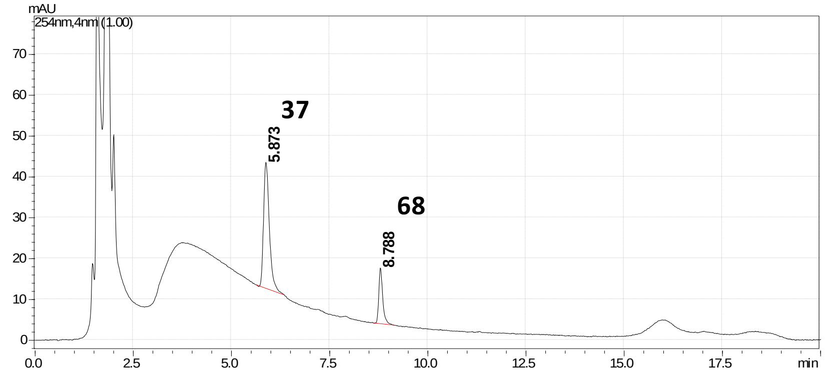

Supplementary Figure 4. Stability of **64** in mouse plasma (substrate concentration: 50 µM, 5% DMSO)

a) t=0 min

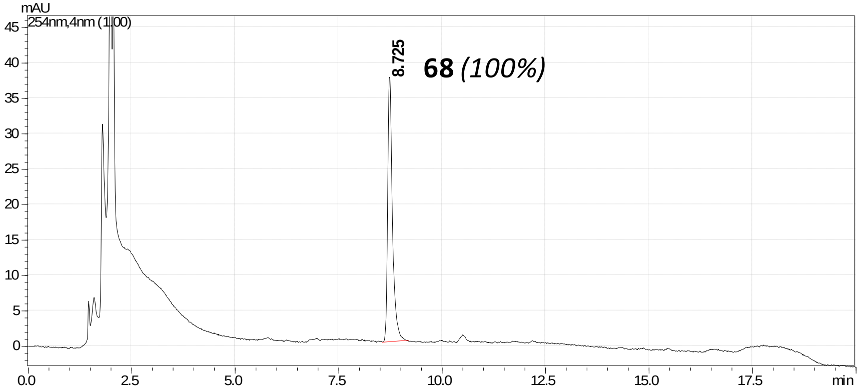

b) t= 30 min

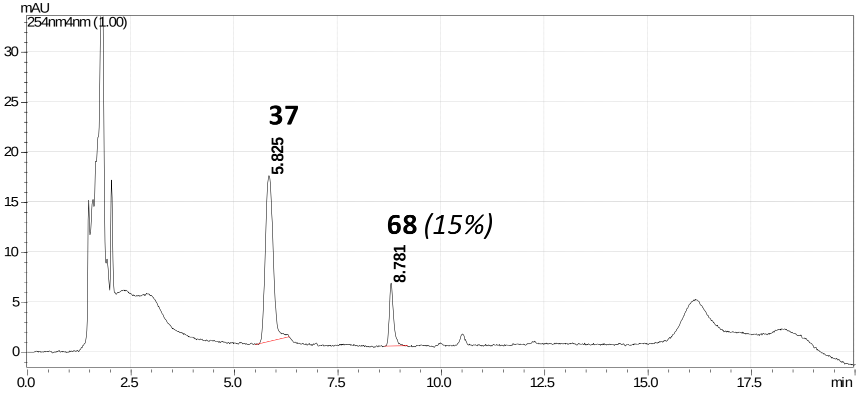

Supplementary Figure 5. Stability of PI3K inhibitors in mouse plasma (substrate concentration: 50 µM, 5% DMSO)

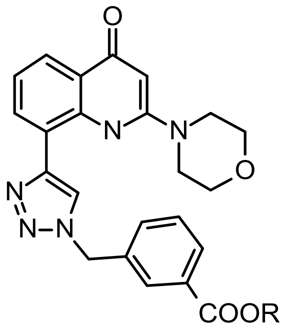

64	R	67	R	66	R
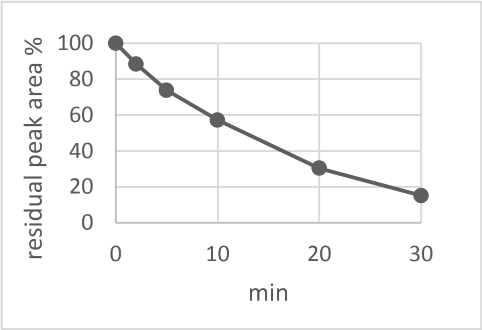	Me	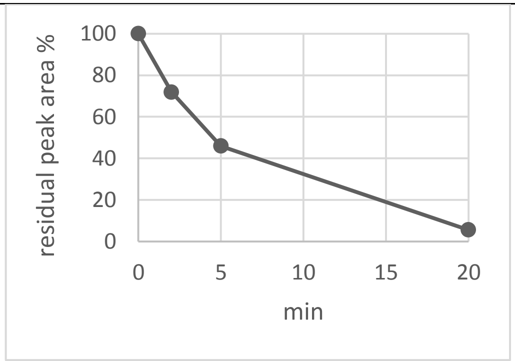	Et	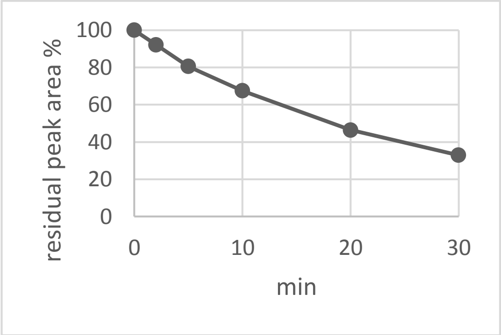	*i*-Pr
**68**	**R**	**69**	**R**	**73**	**R**

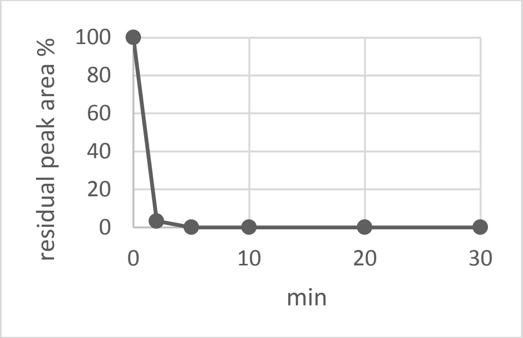

74 R = *n*-Bu

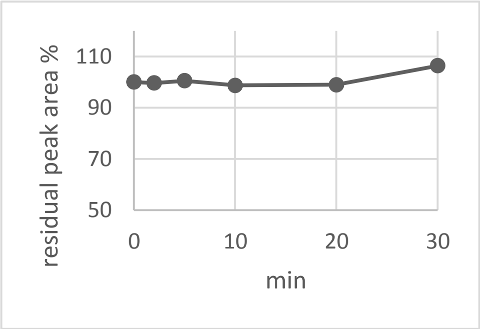

75 R = *t*-Bu

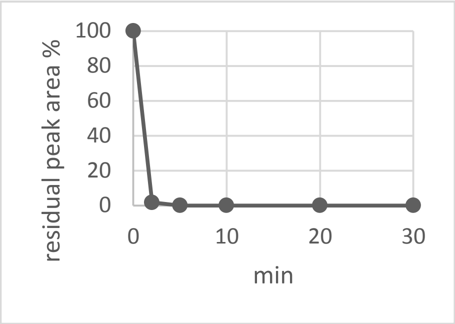

76 R =

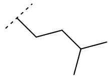

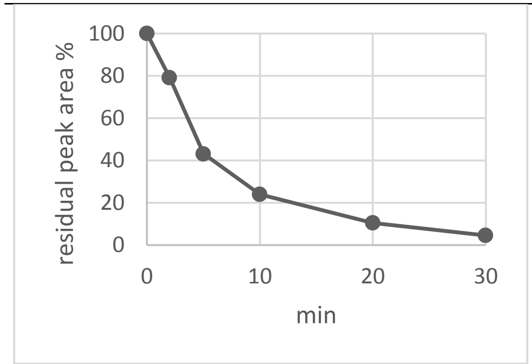

65 R =

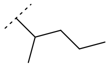

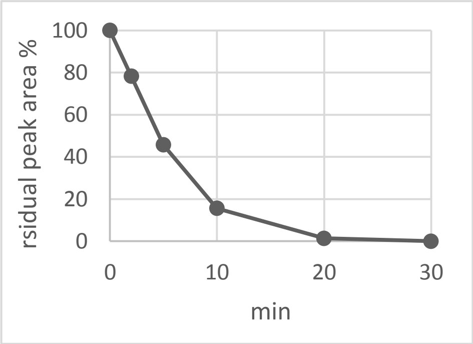

70 R = $C_{11}H_{23}$

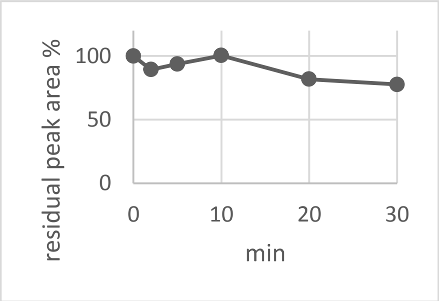

71 R = $C_{18}H_{37}$

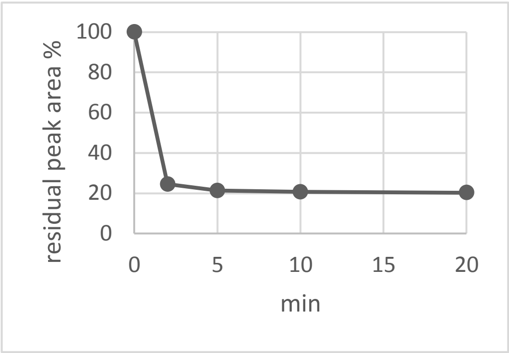
residual peak area %
100
80
60
40
20
0
0
5
10
15
20
min

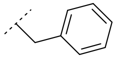

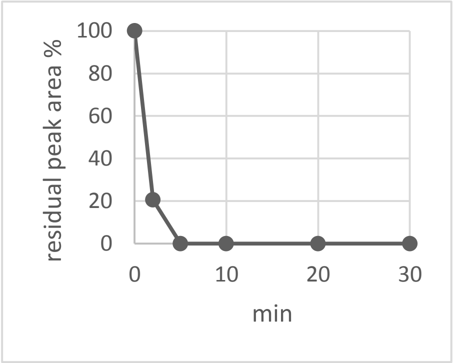
residual peak area %
100
80
60
40
20
0
0
10
20
30
min

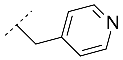
N

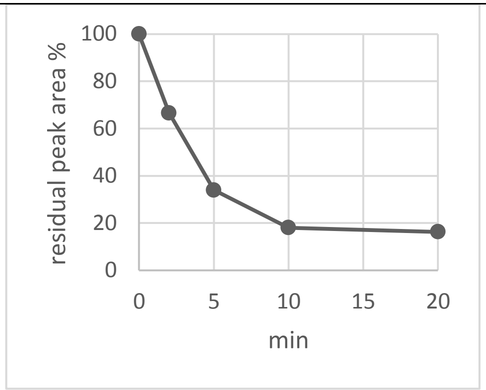
residual peak area %
100
80
60
40
20
0
0
5
10
15
20
min

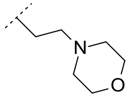
N
O

72 **R**

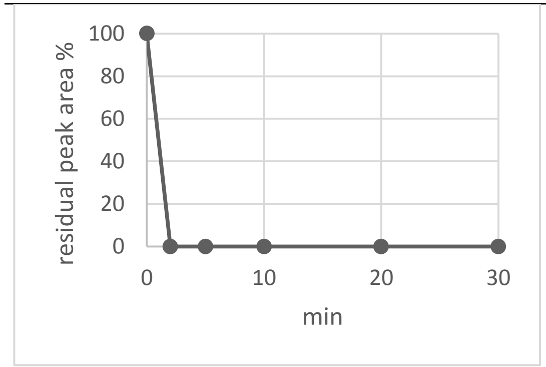
residual peak area %
100
80
60
40
20
0
0
10
20
30
min

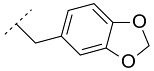
O
O

References

1. Berndt, A.; Miller, S.; Williams; O.; Le, D. D.; Houseman, B. T.; Pacold, J. I.; Gorrec, F.; Hon, W. C.; Liu, Y.; Rommel, C.; Gaillard, P.; Rückle, T.; Schwarz, M. K.; Shokat, K. M.; Shaw, J. P.; Williams, R. L. *Nat Chem Biol.* **2010**, *6*,117-124.
2. Gorrec, F. *J. Appl. Cryst.* **2009**, *42*, 1035-1042.
3. Kabsch, W. XDS. *Acta Cryst.* **2010**, *D66,* 125-132.
4. Collaborative Computational Project, Number 4. *Acta Cryst.* **1994**, *D50*, 760-763.
5. Evans, P. R. *Acta Cryst.* **2006**, *D62*, 72-82.
6. McCoy, A. J.; Grosse-Kunstleve, R. W.; Adams, P. D.; Winn, M. D.; Storoni, L. C.; R. J. Read, R. J.; *J. Appl. Cryst.* **2007**, *40*, 658-674.
7. Adams, P. D.; Afonine, P. V.; Bunkóczi, G.; Chen, V. B.; Davis, I. W.; Echols, N.; Headd, J. J.; Hung, L.-W.; Kapral, G. J.; Grosse-Kunstleve, R. W.; McCoy, A. J.; Moriarty, N. W.; Oeffner, R.; Read, R. J.; Richardson, D. C.; Richardson, J. S.; Terwilliger, T. C.; Zwart, P. H. *Acta Cryst.* **2010**, *D66,* 213-221.
8. Emsley, P.; Cowtan, K. *Acta Cryst.* **2004**, *D60*, 2126-2132.
9. Murshudov, G. N.; Skubak, P.; Lebedev, A. A.; Pannu, N. S.; Steiner, R. A.; Nicholls, R. A.; Winn, M. D.; Long, F.; Vagin, A. A. *Acta Cryst.* **2011**, *D67*, 355-367.
10. Murshudov. G. N.; Vagin, A. A.; Dodson, E. J. *Acta Cryst.* **1997**, *D53*, 240-255. EU Validation contract: BIO2CT-92-0524
